# Genetic Etiology of Parkinson Disease Associated with Mutations in the *SNCA*, *PARK2*, *PINK1*, *PARK7*, and *LRRK2* Genes: A Mutation Update

**DOI:** 10.1002/humu.21277

**Published:** 2010-05-18

**Authors:** Karen Nuytemans, Jessie Theuns, Marc Cruts, Christine Van Broeckhoven

**Affiliations:** 1 Neurodegenerative Brain Diseases Group, Department of Molecular GeneticsVIB, Antwerpen, Belgium; 2 Laboratory of Neurogenetics, Institute Born-Bunge, University of AntwerpAntwerpen, Belgium

**Keywords:** Parkinson disease, genetic etiology, database, *SNCA*, *PARK2*, *PINK1*, *PARK7*, *LRRK2*

## Abstract

To date, molecular genetic analyses have identified over 500 distinct DNA variants in five disease genes associated with familial Parkinson disease; α-*synuclein* (*SNCA*), *parkin* (*PARK2*), *PTEN-induced putative kinase 1* (*PINK1*), *DJ-1* (*PARK7*), and *Leucine-rich repeat kinase 2* (*LRRK2*). These genetic variants include ∼82% simple mutations and ∼18% copy number variations. Some mutation subtypes are likely underestimated because only few studies reported extensive mutation analyses of all five genes, by both exonic sequencing and dosage analyses. Here we present an update of all mutations published to date in the literature, systematically organized in a novel mutation database (http://www.molgen.ua.ac.be/PDmutDB). In addition, we address the biological relevance of putative pathogenic mutations. This review emphasizes the need for comprehensive genetic screening of Parkinson patients followed by an insightful study of the functional relevance of observed genetic variants. Moreover, while capturing existing data from the literature it became apparent that several of the five Parkinson genes were also contributing to the genetic etiology of other Lewy Body Diseases and Parkinson-plus syndromes, indicating that mutation screening is recommendable in these patient groups. Hum Mutat 31:763–780, 2010. © 2010 Wiley-Liss, Inc.

## Introduction

Parkinson disease (PD) is the second most common progressive neurodegenerative brain disorder. It affects 1 to 2% of the population above 65 years and its prevalence increases to approximately 4% in those above 85 years. As these demographic age groups are growing rapidly due to general aging of the population and increasing lifespans, neurodegenerative diseases will represent an ever-growing social and economic burden for society. Through time, the scientific view on PD etiology has changed dramatically. Due to the observation that only 15 to 20% of PD patients have a clear positive family history of PD, researchers predicted that the majority of the PD patients have a complex etiology, including both a genetic and environmental component. During the last 2 decades, molecular genetic analyses in PD families provided important insights in disease mechanisms underlying PD pathology. Nine genes that contribute to the genetic etiology of familial PD were identified through positional cloning strategies in inherited PD patients and families [Bonifati et al., 2003; Di Fonzo et al., 2009; Kitada et al., 1998; Lautier et al., 2008; Paisan-Ruiz et al., 2004, 2009; Polymeropoulos et al., 1997; Ramirez et al., 2006; Valente et al., 2004a; Zimprich et al., 2004a]. Two more PD genes, *UCH-L1* and *HTRA2*, were identified based on the functional relevance of their corresponding protein to PD pathogenesis [Leroy et al., 1998a, b; Strauss et al., 2005]. Although follow-up genetic studies are inconsistent for some of these genes or conclusive data are still pending, ample evidence for a causal association was obtained for PD with five genes, that is, α-*synuclein (SNCA*; MIM] 163890), *parkin (PARK2*; MIM] 602544), *PTEN-induced putative kinase 1 (PINK1*; MIM] 608309), *DJ-1 (PARK7*; MIM] 602533), and *Leucine-rich repeat kinase 2 (LRRK2*; MIM] 609007). Extensive mutation screening of these five causal genes revealed both simple mutations (missense, nonsense, silent, splice site, and untranslated region (UTR) mutations, small insertions and deletions (indels), and copy number variations (CNVs) leading to PD. Approximately 330 confirmed or possible pathogenic mutations in over 1,900 families have been identified so far (Supp. Tables S1-S5; PDmutDB database: http://www.molgen.ua.ac.be/PDmutDB). Possible pathogenic mutations include non-synonymous variants, splice site variants or variants in UTRs that were not observed in control individuals. In this mutation update we present the DNA variants identified so far and elaborate on their clinical and biological relevance. We also discuss the importance of a new publicly available and extensively curated database PDmutDB, and the implications of these analyses for mutation analyses in a diagnostic setting.

## Major Genes and Proteins

### Autosomal Dominant PD Genes

#### α-Synuclein

**Figure 1:**
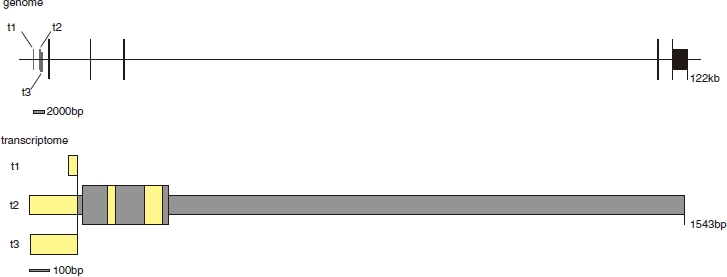
Representation of *SNCA* on genomic and transcript level. All three transcripts coding for the same protein SNCA are depicted (t1: NM 001146055.1 /t2: NM_000345.2/t3: NM_007308.2). On transcript level exons are colored alternately.

*SNCA* was the first causal PD gene identified segregating a pathogenic missense mutation—p.Ala53Thr—in a large Italian family (“Contursi”) (MIM 163890) [Polymeropoulos et al., 1996, 1997] (Table 1 and Fig. 1). The 144aa SNCA protein encoded by the three different *SNCA* transcripts is typically found as a natively unfolded, soluble protein in the cytoplasm or associated with lipid membranes [Davidson et al., 1998] (Table 2). The exact biological function of SNCA in brain is still not fully understood, although there is evidence that implicates SNCA in neurotransmitter release and vesicle turnover at the presynaptic terminals [Abeliovich et al., 2000; Liu et al., 2004].

**Table 1:** Overview of the FiMe Major PD Genes

							Mutation spectrum	
							
Gene	MIM number	Inheritance	Position	Gene size	Number of exons	Transcript length	Classic mutations	Copy number variations
*SNCA*	163890	AD	4q21	112 kb	6	1,543 bp	Missense (0.9%)	Whole gene duplication and triplication (0.6%)
*LRRK2*	609007	AD	12q12	144 kb	51	9,225 bp	Missense (18.2%)	—
*PARK2*	602544	AR	6q26	1.38 Mb	12	4,073 bp	Nonsense, frameshift (indels and splice site), missense (32.4%)	Single or multiple exon deletions and duplications (15.8%)
*PINK1*	608309	AR	1p35-36	18 kb	8	2,660 bp	Nonsense, framshift (indels), missense (24.7%)	Single or multiple exon deletions; whole gene deletion (1.2%)
*PARK7* or *DJ-1*	602533	AR	1p36	34 kb	7	961 bp	Missense (4.4%)	Single or multiple exon deletions and duplications (1.2%)

(%) Number of (possible) pathogenic mutations for this gene/total number of (possible) pathogenic mutations.

**Table 2:** Features of the Proteins Coded by the Five Major Genes

Gene	Protein	Number of aa	Functional domains	(Putative) function
*SNCA*	α-synuclein	144 aa	–	Neurotransmitter release
*LRRK2*	LRRK2	2,527 aa	Ank (ankyrin-like), LRR (leucine rich repeat), Roc (Ras-of-complex proteins), COR (C-terminal of Roc), Kinase, WD40	–
*PARK2*	Parkin	465 aa	UBL (ubiquitin-like), RING1, IBR (in-between-ring), RING2	Target proteins for degradation, maintenance mitochondrial function
*PINK1*	PINK1	581 aa	Target sequence, kinase	Oxidative stress response, maintenance mitochondrial function
*PARK7* or *DJ-1*	DJ-1	189 aa	–	Redox sensor, antioxidant

Mutations in *SNCA* are rather rare and explain disease in ∼2.5% of known unrelated affected carriers (see Supp. Tables S1-1 and S1-2 for mutations, PDmutDB for all references: http://www.molgen.ua.ac.be/PDmutDB). Apart from the Italian Contursi family, p.Ala53Thr was also identified in several families of Greek descent [Athanassiadou et al., 1999; Papadimitriou et al., 1999; Polymeropoulos et al., 1996, 1997; Spira et al., 2001]. More recently, p.Ala53Thr was also detected in two other unrelated families from Asia and Sweden [Choi et al., 2008; Ki et al., 2007; Puschmann et al., 2009] as well as in one seemingly sporadic PD patient of Polish origin [Michell et al., 2005]. With only two other missense mutations identified in *SNCA*—p.Ala30Pro [Kruger et al., 1998] and p.Glu46Lys [Zarranz et al., 2004] (see Supp. Table S1-1)—both also located in the N-terminus of the protein, the missense mutation frequency of *SNCA* in different populations remains very low. In 2003, a triplication of the wild-type *SNCA* locus was observed in a large multigenerational family [Singleton et al., 2003], instigating the discovery of *SNCA* multiplications in several other families with PD and related LBD disorders (see Supp. Table S1-2 for mutations, PDmutDB for all references: http://www.molgen.ua.ac.be/PDmutDB) [Chartier-Harlin et al., 2004; Fuchs et al., 2007; Ibanez et al., 2004, 2009; Ikeuchi et al., 2008; Nishioka et al., 2006, 2009; Nuytemans et al., 2009]. Several of these dosage studies attempted to delineate the boundaries of the multiplicated genomic region identified in families or shared between unrelated carriers. Most *SNCA* multiplicated regions appeared in different genomic sizes (see Supp. Table S1-2), suggestive of independent mutational events. Few studies, however, reported equally sized duplicated or triplicated regions surrounding *SNCA* amongst different families or within branches of the same family [Fuchs et al., 2007; Nishioka et al., 2009]. Of interest is that *SNCA* duplications were also reported in four apparently sporadic PD patients [Ahn et al., 2008; Nishioka et al., 2009; Nuytemans et al., 2009].

#### Leucine-rich repeat kinase 2 or dardarin

**Figure 2:**
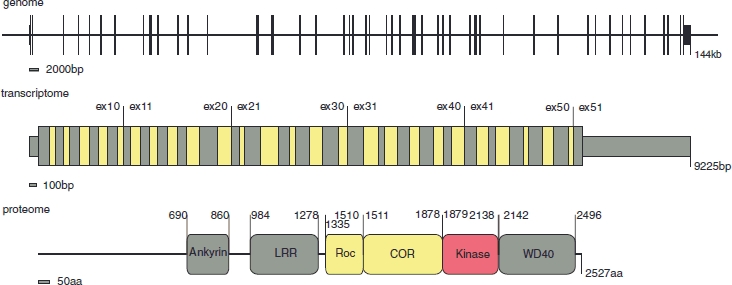
Representation of *LRRK2* on genomic and transcript level and the functional domains of the LRRK2 protein. On transcript level exons are colored alternately (NM_198578.2). (LRR: leucine-rich repeat; Roc: Ras-of-complex protein; COR: C-terminal of Roc.)

The leucine-rich repeat kinase 2 gene *(LRRK2)* was the second causal gene linked to autosomal dominant inherited PD (MIM] 609007) [Funayama et al., 2002; Paisan-Ruiz et al., 2004; Zimprich et al., 2004a, 2004b] (Table 1 and Fig. 2). Its transcript contains 51 exons coding for the LRRK2 protein [Paisan-Ruiz et al., 2004] (Table 2). LRRK2 comprises several functional domains suggestive of on the one hand a kinase activity dependent on the GTPase function of the Roc domain and on the other hand a scaffold protein function implied by the multiple protein–protein interaction regions (Fig. 2). Of interest is that LRRK2 was shown to form dimers under physiological conditions [Greggio et al., 2008]. The exact biological function of LRRK2 remains largely unknown, because no physiological substrates have been identified so far.

The first two publications of PD associated mutations in *LRRK2* described four different pathogenic missense mutations segregating in families of European and North-American origin [Paisan-Ruiz et al., 2004; Zimprich et al., 2004a]. Subsequent mutation analyses identified about 80 discrete missense mutations in over a 1,000 families and sporadic patients worldwide (see Supp. Table S2 for mutations, PDmutDB for all references: http://www.molgen.ua.ac.be/PDmutDB). This corresponds to about 50% of all reported unrelated carriers of mutations in the five major genes, making *LRRK2* the most frequently mutated PD gene so far (Table 3 and PDmutDB: http://www.molgen.ua.ac.be/PDmutDB). The 80 missense mutations are located over the entire LRRK2 protein and affect all predicted functional domains. Some mutations, though, have much higher frequencies than others, for example, p.Gly2019Ser and mutations altering codon Arg1441. Unfortunately, because of the large number of coding exons, only a minority of studies performed mutation analyses of the complete coding region. Most studies focused instead on those exons coding for functional relevant protein domains, namely, Roc, COR, and kinase domains (Fig. 2). Only three studies included dosage analyses aiming at detecting CNVs but did not detect *LRRK2* multiplications or deletions [Mata et al., 2005b; Nuytemans et al., 2009; Paisan-Ruiz et al., 2008]. Nonetheless, rare CNVs of *LRRK2* or parts thereof cannot be excluded, before more dosage studies have been performed for *LRRK2*.

**Table 3:** Relative Frequencies of Mutation Categories Dependent on Ethnicity and Familial History

		*SNCA* (%)		*LRRK2* (%)		*PARK2* (%)			*PINK1* (%)		PARK7 (%)	
						
Ethnic origin		Classic	CNV	Classic	CNV	Classic	mixed	CNV	Classic	CNV	Classic	CNV
Caucasian	F	4.13	2.07	67.36	0	10.12	3.51	7.44	3.93	0.21	0.83	0.41
	S	0.99	0.33	52.48	0	18.15	2.97	11.88	10.89	0.33	0.99	0.66
Asian	F	1.01	8.08	9.09	0	9010	9010	42.42	17.17	0	3.03	0
	S	0	3.13	10.42	0	28.13	1.04	38.54	17.71	1.04	0	0
Arab	F	0	0	88.61	0	1.27	1.27	3.80	3.80	1.27	0	0
	S	0	0	97.06	0	1.47	0	0.74	0	0	0.74	0
Latin-American	F	0	0	57.14	0	14.29	4.76	23.81	0	0	0	0
	S	0	0	41.67	0	41.67	0	8.33	0	8.33	0	0
Ashkenazi Jews	F	0	0	100.00	0	0	0	0	0	0	0	0
	S	0	0	98.04	0	0	0	0	0	0	1.96	0

(%) Number of unrelated mutation carriers with this category of mutation/total number of unrelated mutation carriers (for each ethnicity and familial history). Each row of this table equals 100%.

An important observation is that the *LRRK2* mutation frequency is seemingly dependent on the ethnicity of the population analysed. For example, the most frequent mutation with a strong founder effect—p.Gly2019Ser—was reported worldwide with an average frequency of 1% in PD patients [Paisan-Ruiz, 2009]. But, in Arab Berber and Ashkenazi Jewish populations the p.Gly2019Ser frequency was significantly higher (20 and 40%, respectively) [Lesage et al., 2006; Ozelius et al., 2006], whereas in the first comprehensive screening of a Belgian population, p.Gly2019Ser was apparently absent [Nuytemans et al., 2008]. Other codons in *LRRK2* are also frequently mutated as a consequence of numerous independent mutational events. The p.Arg1441 codon constitutes a mutation hotspot with three different codon substitutions: p.Arg1441Cys, p.Arg1441Gly, and p.Arg1441His. The relatively high mutation frequencies of these mutations should be approached with some caution though, because underlying founder effects have been reported. The most frequent mutation p.Gly2019Ser is observed on a limited number of haplotypes. Also, p.Arg1441Gly was transmitted from a common founder in the Basque population [Gaig et al., 2006; Gonzalez-Fernandez et al., 2007; Gorostidi et al., 2009; Mata et al., 2005c; Paisan-Ruiz et al., 2004; Simon-Sanchez et al., 2006] while p.Arg1441Cys was observed worldwide on several different founder haplotypes [Di Fonzo et al., 2006a; Gaig et al., 2006; Goldwurm et al., 2005; Gosal et al., 2007; Haugarvoll et al., 2008; Hedrich et al., 2006b; Nuytemans et al., 2008; Pankratz et al., 2006a; Tan et al., 2006a]. Additionally, several missense mutations seemed to be (nearly) private mutations for Asian populations: p.Arg1628-Pro, p.Pro755Leu, and p.Gly2385Arg [An et al., 2008; Di Fonzo et al., 2006b; Farrer et al., 2007; Fung et al., 2006b; Ross et al., 2008; Tan et al., 2007, 2008, 2009; Tomiyama et al., 2008].

In contrast to other PD genes, mutations in *LRRK2* have a relatively high frequency of up to 2% in sporadic, late-onset PD patients [Di Fonzo et al., 2005; Gilks et al., 2005; Nichols et al., 2005; Tomiyama et al., 2006]. The high mutation frequency in both familial and sporadic patients makes *LRRK2* the most frequently mutated gene of the five major PD genes. Some prudence in interpreting data is warranted though. Some of the missense mutations have also been reported in healthy control individuals, raising questions on the biological role of these rare variants in disease [Meeus et al., 2010]. The highly variable onset ages associated with *LRRK2* mutations [Hernandez et al., 2005; Kachergus et al., 2005; Paisan-Ruiz et al., 2005; Zimprich et al., 2004a], the presence of *LRRK2* mutations in unaffected individuals [Carmine Belin et al., 2006; Di Fonzo et al., 2006a; Gaig et al., 2006; Hernandez et al., 2005; Kay et al., 2005; Khan et al., 2005b; Latourelle et al., 2008; Nichols et al., 2005; Zimprich et al., 2004a], and the high frequency in sporadic patients render the assessment of pathogenicity of the identified variants extremely difficult as these issues complicate segregation analyses. To date, pathogenicity supported by segregation analyses has only been demonstrated for six *LRRK2* mutations (p.Arg1441Cys, p.Arg1441Gly, p.Tyr1699Cys, p.Gly2019Ser, and p.Ile2020Thr).

### Autosomal recessive PD genes

#### PARK2 or parkin

**Figure 3:**
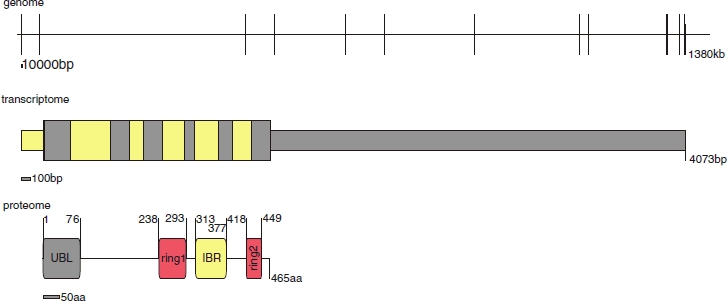
Representation of *PARK2* on genomic and transcript level and the functional domains of the parkin protein. On transcript level exons are colored alternately (NM_004562.2). (UBL: ubiquitin-like; IBR: in-between-ring.)

The first of three recessive PD genes identified is *PARK2* (MIM 602544), which was linked with disease in a nuclear Japanese consanguineous family [Kitada et al., 1998] (Table 1 and Fig. 3). *PARK2* spans approximately 1.38 Mb and encodes the protein parkin. The 456 amino acid protein harbors four major functional domains corresponding to its function as an E3 ubiquitin ligase (Table 2) [Imai et al., 2000; Shimura et al., 2000; Zhang et al., 2000]. Its role in the ubiquitin proteasome system (UPS) comprises of tagging dysfunctional or excessive proteins for degradation. Further, it was shown that under physiological conditions parkin is involved in mitochondrial maintenance [Deng et al., 2008a; Exner et al., 2007; Park et al., 2009; Poole et al., 2008; Weihofen et al., 2009] and might induce subsequent autophagy of dysfunctional mitochondria [Narendra et al., 2008, 2009].

The first mutation reports indicated a wide spectrum of loss-of-function mutations in *PARK2* including simple mutations like nonsense, missense and splice site mutations, indels, as well as CNVs of the promoter region and single or multiple exons (Table 2) [Hattori et al., 1998a, b; Kitada et al., 1998]. *PARK2* mutations were identified spread across the entire gene in either homozygous, compound heterozygous or heterozygous state in familial and sporadic patients from different ethnicities (see Supp. Table S3-1 and S3-2 for mutations, PDmutDB for all references: http://www.molgen.ua.ac.be/PDmutDB). Heterozygous *PARK2* variants have also been observed in healthy control individuals, making assessment of pathogenicity for these variants quite complex. Approximately 40% of unrelated mutation carriers were reported to harbor a mutation in *PARK2* (Table 3 and PDmutDB: http://www.molgen.ua.ac.be/PDmutDB). Of these, close to 8% carry both a simple mutation as a CNV, whereas carriers of only simple mutations or CNVs are almost equally common (43.8% vs. 47.9%). Investigation of the haplotypes on which frequent *PARK2* mutations reside, showed that most CNVs are independent events, whereas point mutations were more commonly transmitted from common founders [Periquet et al., 2001]. This suggests that the high mutation frequency in *PARK2* is only partly due to small founder effects.

#### P-TEN-induced putative kinase 1

**Figure 4:**
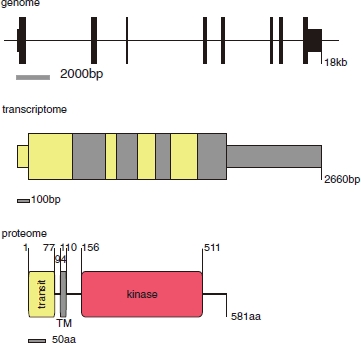
Representation of *PINK1* on genomic and transcript level and the functional domains of the PINK1 protein. On transcript level exons are colored alternately (NM_032409.2). (TM: transmembranair.)

Homozygosity mapping in *PARK2* negative European families led to the identification of the second autosomal recessive gene, *P-TEN induced putative kinase 1 (PINK1*; MIM] 608309) [Valente et al., 2001, 2002, 2004a] (Table 1 and Fig. 4). The PINK1 protein is a putative serine/threonine kinase involved in mitochondrial response to cellular and oxidative stress [Valente et al., 2004a] (Table 2). This response is likely mediated by regulation of the calcium efflux, influencing processes such as mitochondrial trafficking [Wang and Schwarz, 2009; Weihofen et al., 2009], ROS formation, mitochondrial respiration efficacy [Liu et al., 2009], and opening of the mitochondrial permeability transition pore [Gandhi et al., 2009] as well as by interaction with cell death inhibitors and chaperones [Plun-Favreau et al., 2007; Pridgeon et al., 2007; Wang et al., 2007]. In addition, PINK1 is an important player in the alleged PINK1/parkin pathway, regulating mitochondrial morphology and functionality in response to stressors [Deng et al., 2008a; Exner et al., 2007; Park et al., 2009; Poole et al., 2008; Weihofen et al., 2009].

The *PINK1* mutation spectrum involves nonsense and missense mutations, indels, and whole-gene or single/multiple exon CNVs (Table 2) located across the entire gene. Mutation analyses in familial as well as sporadic patients identified homozygous and compound heterozygous mutations (see Supp. Table S4-1 for mutations, PDmutDB for all references: http://www.molgen.ua.ac.be/PDmutDB). Approximately 6.5% of known mutation carriers carry a mutation in *PINK1* (Table 3). Again, many putative pathogenic mutations were also observed in heterozygous state in familial and sporadic patients as well as in healthy control individuals [Abou-Sleiman et al., 2006; Bonifati et al., 2005; Brooks et al., 2009; Choi et al., 2008; Djarmati et al., 2006; Fung et al., 2006a; Healy et al., 2004; Klein et al., 2005; Kumazawa et al., 2008; Mellick et al., 2009; Nuytemans et al., 2009; Rogaeva et al., 2004; Tan et al., 2005, 2006b; Valente et al., 2004b; Weng et al., 2007]. With the current available mutation data, it seems that CNVs in *PINK1* are less common than simple loss-of-function mutations (see Supp. Table S4-2). But at this stage we cannot exclude that this observation represents an ascertainment bias because many studies did not perform *PINK1* dosage analyses and therefore might have missed CNVs in their patient groups.

#### PARK7 or DJ-1

**Figure 5:**
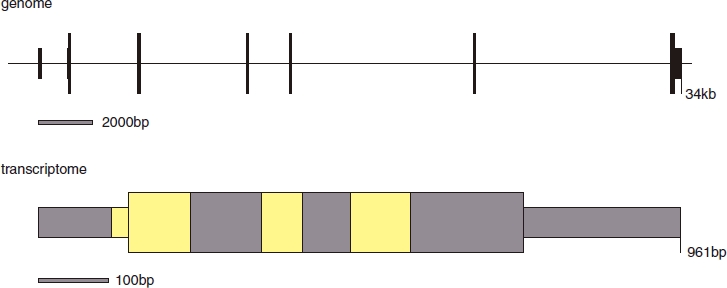
Representation of *PARK7* on genomic and transcript level. On transcript level exons are colored alternately (NM_007262.4).

The third autosomal recessive PD gene, *PARK7* (or *DJ-1*; MIM 602533) was identified by homozygosity mapping in an extended Dutch family with multiple consanguinity loops [Bonifati et al., 2003; van Duijn et al., 2001] (Table 1 and Fig. 5). The DJ-1 protein was found to be H_2_O_2_ responsive suggesting that DJ-1 represents a sensor for oxidative stress, for example, dopamine toxicity [Lev et al., 2009], and acts as an antioxidant [Mitsumoto and Nakagawa, 2001] (Table 2). It was further hypothesized that DJ-1 could be part of a novel E3 ligase complex together with parkin and PINK1 [Xiong et al., 2009].

Mutation analyses identified homozygous, compound heterozygous as well as heterozygous [Bonifati et al., 2003; Clark et al., 2004; Hague et al., 2003; Hedrich et al., 2004a; Nuytemans et al., 2009] missense mutations and CNVs in patients (see Supp. Tables S5-1 and S5-2 for mutations, PDmutDB for all references: http://www.molgen.ua.ac.be/PDmutDB). Also for *PARK7*, heterozygous variants were observed in control individuals. Mutations in *PARK7* are reported near 1% of all known mutation carriers (Table 3). Current mutation data indicates that CNVs in *PARK7* are less frequent than simple mutations. But, because of the rarity of mutations in *PARK7*, most studies have not analysed their PD patient groups, making it highly likely that putative pathogenic mutations have been missed and that the current mutation frequency of *PARK7* is an underestimate.

## Clinical Implications

Clinical features of PD patients typically include tremor, bradykinesia, rigidity, good levodopa response, and/or postural instability. Interestingly, PD is part of a wide Lewy Body Diseases (LBD) spectrum made up by closely related clinical phenotypes characterized by variable manifestation of parkinsonism and dementia (PD, PD with dementia [PDD], Dementia with LB (DLB), LB variant of Alzheimer's disease [AD] and AD). On the other hand, parkinsonism can be accompanied by additional atypical features defining the parkinson-plus syndromes, like multiple system atrophy (MSA; dysautonomia and/or cerebellar signs), progressive supranuclear palsy (PSP; impaired vertical eye movements and prominent postural instability) and corticobasal degeneration (CBD; apraxia). The clinical features reported in literature are mostly typical for disorders of the LBD spectrum. In some cases, however, more atypical features indicative of other related diseases, such as the Parkinson-plus syndromes were observed. This indicates there is a high variability in phenotypes associated by mutations in *SNCA, LRRK2, PARK2, PINK1*, and *PARK7*.

Here we summarize typical and atypical presentations of specific mutation groups and discuss some of its implications. This and more detailed information on familial, individual, and clinical data can be found in the newly constructed and publicly available PDmutDB database (http://www.molgen.ua.ac.be/PDmutDB).

*SNCA* is the only one of the five genes in which an obvious correlation can be made between distinct missense mutations or distinct CNVs and the resulting different phenotypes. The majority of the familial PD patients carrying the SNCA missense mutations p.Ala53Thr or p.Ala30Pro typically present with bradykinesia and rigidity at an early onset age (< 55years) [Bostantjopoulou et al., 2001; Ki et al., 2007; Kruger et al., 1998; Papapetropoulos et al., 2001, 2003; Puschmann et al., 2009; Spira et al., 2001]. The sporadic Polish patient carrying p.Ala53Thr, however, showed typical PD features, that is, late onset at 74 years, rigidity, progressive bradykinesia, and mild tremor [Michell et al., 2005]. Also, clinical features in carriers of the third SNCA missense mutation p.Glu46Lys are atypical in such that these carriers present with symptoms at later age and suffer from dementia within several years after PD onset [Zarranz et al., 2004]. Brain pathology in one p.Glu46Lys carrier showed diffuse LB consistent with a diagnosis of DLB confirming the atypical clinical presentation [Zarranz et al., 2004]. Also, patients carrying *SNCA* multiplications present with atypical forms of the disease. A direct correlation between phenotype and number of *SNCA* copies was consistently observed among different studies. Most duplication carriers present with late-onset parkinsonism [Chartier-Harlin et al., 2004; Fuchs et al., 2007; Ibanez et al., 2004, 2009; Nishioka et al., 2006, 2009], which can be accompanied by a later onset cognitive decline (PDD) [Nishioka et al., 2006, 2009; Nuytemans et al., 2009]. Triplication carriers however seem to be more severely affected suffering from a more aggressive form of dementia despite their shorter disease duration (DLB) [Farrer et al., 2004; Ibanez et al., 2009; Singleton et al., 2003]. Also, asymptomatic carriers have been reported in families of both seemingly sporadic and familial PD patients [Ahn et al., 2008; Ibanez et al., 2009; Nishioka et al., 2006, 2009]. Only few of these carriers have exceeded the onset age of the proband [Ibanez et al., 2009; Nishioka et al., 2009], indicating variable onset ages or reduced penetrance for this mutation. When considering all unaffected duplication carriers that are older than the average onset age of the affected carriers as true asymptomatic individuals a crude estimate of 85% penetrance could be obtained from the information in PDmutDB (http://www.molgen.ua.ac.be/PDmutDB). Interestingly, one study describing both duplication and triplication of *SNCA* in two separate branches of the same family, also reported clinical features reminiscent of MSA (orthostatic hypotension and poor levodopa response) in both branches [Fuchs et al., 2007].

Typically, patients carrying *LRRK2* missense mutations present with clinical features similar to those of idiopathic PD, that is, asymmetrical late onset, bradykinesia, rigidity, tremor, and good l-dopa response. The incidence of tremor, however, seems to be elevated in *LRRK2* carriers indicating that *LRRK2* mutations most likely lead to tremor-dominant disease [Haugarvoll et al., 2008; Nuytemans et al., 2008; Paisan-Ruiz et al., 2004]. On the other hand, isolated studies have also reported *LRRK2* mutations in carriers with a clinical diagnosis of sporadic PD with late-onset AD as well as CBD, PSP, or frontotemporal dementia (FTD) [Chen-Plotkin et al., 2008; Santos-Reboucas et al., 2008; Spanaki et al., 2006].

Clinical features of *PARK2* homozygous mutation carriers are generally indistinguishable from those of idiopathic PD patients with the exception of a clear drop in onset age. Typically *PARK2* patients present with disease onset before the age of 50 years and a slow disease progression [Abbas et al., 1999; Khan et al., 2005a; Lucking et al., 2000]. Although they respond well to levodopa treatment they are more likely to develop treatment-induced motor complications earlier in the treatment [Deng et al., 2008b; Khan et al., 2005a; Lucking et al., 2000]. Further, *PARK2* mutations were also identified in patients with a clinical diagnosis of PSP, PD plus essential tremor (ET), as well as ET and restless legs syndrome (RLS) [Adel et al., 2006; Deng et al., 2007; Limousin et al., 2009; Pellecchia et al., 2007; Pigullo et al., 2004; Sanchez et al., 2002].

Homozygous *PINK1* mutation carriers are clinically indistinguishable from homozygous *PARK2* mutation carriers [Bentivoglio et al., 2001; Valente et al., 2004a]. Although rare, *PINK1* mutations were also associated with late-onset PD, RLS with parkinsonism, and dopa-responsive dystonia [Gelmetti et al., 2008; Leutenegger et al., 2006; Tan et al., 2005, 2006b]. Further, a few *PINK1* homozygous mutation carriers also presented with cognitive and psychiatric problems in addition to parkinsonism [Ephraty et al., 2007; Reetz et al., 2008; Savettieri et al., 2008].

Clinical features of carriers with a homozygous mutation in the recessive *PARK7* gene are also similar to those of homozygous *PARK2* and *PINK1* carriers [Bonifati et al., 2003]. Also here, clinical heterogeneity with a wide range of clinical phenotypes among unrelated and related carriers was reported. For example, in one family segregating two distinct homozygous variations were diagnosed with early onset parkinsonism, dementia, and amyo-trophic lateral sclerosis (ALS) [Annesi et al., 2005]. In addition, the initially reported 14 kb deletion of the 5^0^ region of *PARK7* linked to typical PD was also observed heterozygously in two dementia patients without signs of parkinsonism [Arias et al., 2004].

The available clinical data showed us that mutations in these five PD genes are not only present in patients but also in patients diagnosed with related disorders. Some clinical features are known to overlap between these disorders, so clinical diagnoses may not always be accurate or different disorders might share a common etiology. In both cases, it might be worthwhile screening for mutations in “PD-associated-genes” in larger groups of patients with clinical diagnoses related to PD to further explore the genotype-phenotype correlations. Alternatively, no information was provided on mutation analyses of additional genes so other currently unknown mutations might still explain this range of clinical features for these patients.

When discussing genotype-phenotype correlations, one needs to take into account that at times it can be difficult to comprehend the clinical implications of some genetic variants. Although homozygous mutations in the recessive genes have a penetrance of 100% with only two carriers older than the onset age of affected relatives reported in literature (*PARK2* p.Trp74fsCysX8 [Pineda-Trujillo et al., 2001] and *PARK7* p.Glu64Asp [Hering et al., 2004]), the effect of heterozygous mutations is far less clear. The presence of these mutations in *PARK2, PINK1*, and *PARK7* has instigated a debate on the role of heterozygous recessive mutations as risk factors for disease. In many studies the prevalence of these heterozygous rare variants is (significantly) higher in patients than in control individuals (*PARK2*: [Brooks et al., 2009; Clark et al., 2006; Lesage et al., 2008; Nuytemans et al., 2009; Sun et al., 2006]/*PINK1*: [Abou-Sleiman et al., 2006; Bonifati et al., 2005; Brooks et al., 2009; Marongiu et al., 2008; Rogaeva et al., 2004; Valente et al., 2004b]), implying that the presence of a heterozygous recessive mutation might increase the carrier's susceptibility to develop PD. In addition, several families reported affected heterozygous family members of a homozygous proband creating a false impression of dominant inheritance and indicating a possible predisposition of *PARK2* or *PINK1* variants to PD (*PARK2*: [Maruyama et al., 2000; Munhoz et al., 2004; Tan et al., 2003]/*PINK1*: [Criscuolo et al., 2006; Djarmati et al., 2006; Hedrich et al., 2006a; Ibanez et al., 2006]). Investigation of clinical features in patients with digenic combinations of heterozygous mutations might provide us with more insight in the effects of these variants (Table 4). For example, the clinical presentation and onset of PD does not differ between patients carrying a heterozygous *LRRK2* mutation and patients carrying a digenic combination of *LRRK2* and *PARK2* mutations [Bras et al., 2008; Ferreira et al., 2007; Gao et al., 2009; Illarioshkin et al., 2007; Lesage et al., 2006; Marras et al., 2010]. Illiaroshkin and coworkers, though, reported early occurrence of dyskinesias during treatment, more common in *PARK2* mutation carriers, in a *LRRK2/PARK2* digenic mutation carrier [Illarioshkin et al., 2007]. Reports of carriers with digenic mutations of two recessive genes are rare, mostly because many mutation studies reported so far have not analyzed all five PD genes. One study describing patients carrying a single heterozygous *PINK1* mutation on top of a homozygous *PARK2* mutation indicated, nevertheless, that these patients present with a significant earlier onset age than patients carrying only *PARK2* mutations [Funayama et al., 2008]. This suggested that heterozygous *PINK1* mutations might indeed effect the development of PD, although more research into their biological role is warranted.

**Table 4:** Relative Frequencies of Homozygotes or Compound Heterozygotes and Digenic Combinations Dependent on Ethnicity and Familial History

Ethnic origin			Homozygotes (%)	Compound heterozygotes (%)	Digenic combinations (%)		
Caucasian	F	10.33	*LRRK2, PARK2, PINK1*, and *DJ-1*	8.06	*LRRK2, PARK2*, and *PINK1*	0.20	*LRRK2-PARK2*
	S	8.58	*PARK2, PINK1*, and *DJ-1*	6.60	*LRRK2, PARK2*, and *PINK1*	1.65	*LRRK2-PARK2*
Asian	F	41.41	*SNCA, PARK2, PINK1*, and *DJ-1*	22.22	*PARK2* and *PINK1*	1.01	*PINK1-DJ-1*
	S	38.54	*PARK2*, and *PINK1*	6.25	*PARK2*	0	
Arab	F	50.63	*LRRK2, PARK2*, and *PINK1*	1.27	*PARK2*	0	
	S	13.97	*LRRK2, PARK2*, and *DJ-1*	0		0	
Latin-American	F	23.81	*PARK2*	14.29	*PARK2*	0	
	S	16.67	*LRRK2* and *PINK1*	25.00	*PARK2*	0	

(%) Number of unrelated mutation carriers with this category of mutation/total number of unrelated mutation carriers (for each ethnicity and familial history).

## Biological and Pathological Relevance

The pathology in PD brain generally consists of progressive neuronal depigmentation and dopaminergic cell loss in the substantia nigra, accompanied by presence of LB in the residual neurons (Table 5). Interestingly, the LB are common to all disorders in the LBD spectrum, although their location in the patient's brain can help specify the exact disorder. In nondemented PD patients the LB are usually confined to the brainstem, whereas more widespread cortical LB point to PDD or DLB. It is not fully understood yet how mutations in the causal PD genes might cause such pathology. Because SNCA is the main constituent of LB [Baba et al., 1998], many studies have tried elucidating the biological processes that trigger SNCA aggregation. Direct investigation of SNCA itself has provided evidence that mutant SNCA has a greater tendency to acquire a misfolded conformation [Conway et al., 2000; Cookson, 2005; Kazantsev and Kolchinsky, 2008], stabilized by oligomerisation [Uversky et al., 2001a, b]. But overexpression of wild-type SNCA produces the same effect by triggering a shift from natively unfolded SNCA to small oligomers due to concentration burden [Kazantsev et al., 2008; Uversky et al., 2001b]. Aggregation of SNCA has been shown to be neurotoxic for the cell through the formation of intermediate aggregates called protofibrils [Conway et al., 2000; Spillantini et al., 1998]. Because of their conformation these protofibrils can bind lipid membranes and cause membrane permeabilization. It is suspected that LB sequester these protofibrils as part of a defense mechanism of the cell against toxic effects [Bodner et al., 2006; Kazantsev and Kolchinsky, 2008]. Although a few studies reported the presence of LRRK2 in ubiquitin-positive inclusion bodies [Greggio et al., 2006; Perry et al., 2008], it is generally perceived that LRRK2 does not reside in LB in affected brains. Interestingly though, associated LRRK2 pathology comprises variable lesions; (diffuse) LBs and/or PSP-like tau aggregation or none of the above [Zimprich et al., 2004a], suggesting that LRRK2 dysfunction might be an upstream event in neurodegeneration and causing disturbances in different pathways. The biological function of LRRK2, however, is still largely unknown. Mutations in the kinase domain of LRRK2 (i.e., p.Gly2019Ser and p.Ile2020Thr) were reported to increase kinase activity [Anand et al., 2009; Gloeckner et al., 2006; Greggio et al., 2006; Guo et al., 2007; Imai et al., 2008; West et al., 2005, 2007], but these results were based on autophosphorylation or phosphorylation of heterologous substrates, warranting caution in interpreting these data. The mutations in the Roc domain, the GTPase regulating the kinase domain, are suspected to impair the function of the GTPase, therefore inducing sustained kinase activity of LRRK2 [Guo et al., 2007; Lewis et al., 2007; Li et al., 2007]. Furthermore, mutations like the substitutions at codon p.Arg1441 and p.Arg1442Pro are located at key positions for the formation of functional LRRK2 dimers; possibly also resulting in a decreased GTPase activity [Gotthardt et al., 2008]. As the exact functions of the other domains in LRRK2 in relation to kinase activity are unclear, it is difficult to assess the impact of mutations in these domains.

**Table 5:** Overview of Pathology Associated with Mutations in the Five Different PD Genes

Gene	Pathology	Reference(s)
*SNCA*	Typical LB disease	[Spira et al., 2001]
	Brainstem and cortical LB and neuritic staining	[Farrer et al., 2004; Fuchs et al., 2007; Gwinn-Hardy et al., 2000; Ikeuchi et al., 2008; Obi et al., 2008; Wakabayashi et al., 1998; Waters and Miller, 1994; Zarranz et al., 2004]
*LRRK2*	Typical LB disease	[Giasson et al., 2006; Giordana et al., 2007; Papapetropoulos et al., 2006]
	Tau-positive pathology without LB	[Gaig et al., 2007; Rajput et al., 2006; Zimprich et al., 2004a]
	Nigral degeneration, with neither LB nor NFT	[Dachsel et al., 2007; Gaig et al., 2008; Giasson et al., 2006]
*PARK2*	Loss of dopaminergic neurons in SN and LC without LB or NFT pathology	[Gouider-Khouja et al., 2003; Hayashi et al., 2000; Kitada et al., 1998; Sasaki et al., 2004]
	Typical LB disease	[Pramstaller et al., 2005]
*PINK1*	Typical LB disease	[Samaranch et al., 2010]
*PARK7* or *DJ-1*	Remains to be determined	

LB, lewy body; NFT, neurofibrillary tangles; SN, substantia nigra, LC, locus ceruleus.

The proteins encoded by the recessive PD genes are all involved in the cell's response mechanism to cellular and oxidative stress, implying cell dysfunction or increased vulnerability to neurode-generation in patients carrying mutations in these genes. Mutations in *PARK2* were reported to impair the E3 ubiquitin ligase activity of parkin [Shimura et al., 2000], which resulted in insufficient protein clearance and the subsequent formation of protein aggregates. On the other hand, *PINK1* mutations were shown to interfere with its protein stability and kinase activity [Sim et al., 2006], possible causing disrupted mitochondrial trafficking [Wang and Schwarz, 2009; Weihofen et al., 2009], reduced performance of the electron transport complexes [Gandhi et al., 2009; Liu et al., 2009] and elevated ROS formation [Gandhi et al., 2009] due to disturbed calcium homestasis, as well as activation of cell death proteins [Plun-Favreau et al., 2007; Pridgeon et al., 2007; Wang et al., 2007]. Together parkin and PINK1 are thought to be involved in the same pathway upstream of the mitochondrial fission/fusion machinery and mutations in both have been shown to result in an increase of mitochondrial fission in mammalian cells [Exner et al., 2007; Weihofen et al., 2009]. In addition, parkin was shown to be recruited to dysfunctional mitochondria pointing toward a possible role of parkin in the induction of mitophagy [Narendra et al., 2008, 2009]. Mutations in *PARK2* might impair this function and eventually result in increased cellular toxicity. This hypothesis was supported by a parkin null *Drosophila* model, which showed mitochondrial defects and elevated oxidative stress rather than UPS impairment [Greene et al., 2003; Pesah et al., 2004], implying that parkin's involvement in mitochondria might be its primary activity. Nonetheless, more studies are needed to investigate the contribution of parkin to this pathogenic pathway.

In light of this, it seems plausible that digenic combinations of heterozygous mutations in *PARK2* and *PINK1* could be sufficient to cause disease as they might enhance each other's pathogenic effect by concomitant partial loss of function of two important enzymes active in the same pathway. Further, mutations in *PARK7* were suspected to contribute to neuronal death through loss of antioxidant activity of DJ-1 and subsequent increase in oxidative stress of the cell [Moore et al., 2003; Ramsey and Giasson, 2008; Taira et al., 2004]. It is not clear yet how the *PARK7* mutations can lead to this impaired functionality. As concomitant deficits in mitochondrial function and UPS activity have been observed, one might suspect a feedback loop between both cellular processes ultimately resulting in cell death and protein excess and aggregation.

## Diagnostic Relevance

The past decade has been very exciting for molecular genetic research of PD. Genetic variants in at least 11 genes have been associated with increased risk for PD, and study of the corresponding proteins has been critical for our knowledge of the disease mechanisms underlying PD pathogenesis. For five genes there is extensive evidence of causality but screening all five of them for diagnostic or research purposes is a laborious undertaking. Therefore, mutation studies have been often restricted to sequence analyses of the two most frequently mutated genes—*LRRK2* and *parkin*—and sometimes even further restricted to sequences coding for functional domains within these genes. Therefore, we have incomplete data to calculate the precise contribution of mutations in different PD genes together with an underestimation of more complex mutations like CNVs.

Ideally mutation analyses of the five major PD genes should include both sequence analysis and dosage analyses to detect CNVs. We investigated 310 Flanders-Belgian patients [Nuytemans et al., 2009], and showed high frequencies of heterozygous variants in *PARK2* (9.0%) and *LRRK2* (6.1%) and low contributions for *SNCA, PINK1*, and *PARK7* mutations (0.3, 0.3, and 0.6%, respectively). In contrast to other populations, we did not observe the most frequent mutation in *LRRK2*, p.Gly2019Ser.

It is difficult to compare mutation frequencies between patient groups of different ethnic background because even the more recent and extensive mutation analyses in Brazilian, Dutch, Korean, Australian, or Portugese patient groups [Aguiar et al., 2008; Bras et al., 2008; Camargos et al., 2009; Choi et al., 2008; Macedo et al., 2009; Mellick et al., 2009] employed different study setups (selection of patients, genes of interest, domains of interest, etc.). Here, we provided a comprehensive presentation of the mutation frequencies, based on the published studies (Tables 3 and 4 PDmutDB: http://www.molgen.ua.ac.be/PDmutDB). When analyzing these data it became clear that, as in our Flanders-Belgian study, the contributions of *SNCA, PINK1*, and *PARK7* are relatively low. Remarkably, the mutation burden of *PINK1* was increased almost twofold in Asian patient groups. *LRRK2* remains the most frequently mutated gene, even when heterozygous *PARK2* mutation carriers were included in the equation. These data reinforced the guidelines on molecular diagnosis of PD that were proposed by the European Federation of Neurological Sciences (EFNS) [Harbo et al., 2009]. It is important to stress that the frequency data depicted here were extracted from reported studies only, and therefore is likely biased because *SNCA, PINK1*, and *PARK7* analyses were often incomplete or even absent.

Influences of ethnicity on mutation frequencies as well as founder effects have been documented for several PD genes. Consequently, only a complete mutation analysis of these genes will allow the identification of all relevant mutations both for the individual patient as well as a population of interest. In addition, it is important to go on with the genetic characterization of patients even if they have been shown to carry a mutation in one gene, because unexpected digenic combinations might explain some atypical clinical presentations of individual PD patients. Despite the fact that not all studies implemented CNV analyses the observed CNV frequencies are higher than expected, implying that gene dosage is a major feature of the genetic etiology of PD. For *PARK2*, for instance, in approximately 50% of mutation carriers, deletions or duplications of (single) exons were identified. For *SNCA*, multiplications were observed not only in familial (∼88%) but also in seemingly sporadic patients (∼12%), resulting in higher CNV frequencies than originally anticipated. These data indicate that dosage analysis should be considered in all mutation screenings. On the other hand, when performing extensive mutation analyses, problems with pathogenicity assessment can occur for some types of mutations. For example, genetic variants appearing in *LRRK2* domains with unclear biological function or heterozygous *PARK2* mutations. The current efforts aiming at developing novel functional assays should be helpful in obtaining sufficient evidence to support a pathogenic role—and thus clinical implication—of individual mutations in the near future.

## A PD Mutation Database (PDmutDB)

A huge amount of information on genetic variability in *SNCA, PARK2, PINK1, PARK7*, and *LRRK2* and corresponding clinical phenotypes is present in the scientific literature, though, contained within numerous articles published over the last 2 decades. In addition, the data provided is often incomplete, fragmented, or sometimes even hard to interpret because, for example, clinical and genetic data of one family or group of patients are reported in separate articles and/or in different formats. Some of the current mutation databases do not systematically provide information on clinical features, familial history, and so on, whereas others are maintained by the goodwill of the researchers themselves, and consequently, are often incomplete or not up to date. Therefore, we decided to construct a novel PD mutation database, called PDmutDB (http://www.molgen.ua.ac.be/PDmutDB). This database will be publicly available and will hold information of reported variants with correct nomenclature and references to original studies. To allow for genotype-phenotype correlations, we added detailed familial and clinical data. Importantly, all informative family members are linked to their individual clinical features and identified variants in multiple genes with indication of zygosity. Also, we make an effort to provide an indication of pathogenicity for each variant, whenever sufficient data are available.

Data from new publications will be included in the database whenever they contain sufficient genetic information to correctly link each individual to the respective mutations. Individual researchers can submit genetic and/or clinical information using an additional file when their publications do not permit excessive tables. Contact: PDmutDB@molgen.vib-ua.be.

## Conclusion and Future Prospects

During the last 2 decades molecular genetic research has lead to the identification of five important PD genes bearing approximately 500 different DNA variants. These variants make up a wide mutation spectrum including different simple mutations as well as genomic rearrangements. Gathering this information from literature is very laborious because it is scattered across many publications and different studies employ different study designs. Here we present a novel publicly available mutation database PDmutDB (http://www.molgen.ua.ac.be/PDmutDB). Next to the systematic organization of all DNA variants, this database provides information on family history, clinical features, and mutation zygosity. At this time, data on approximately 1,900 families of sporadic and familial patients are available. Data meta-analysis indicated both high genetic and clinical heterogeneity among mutation carriers. Mutations have been identified in patients with PD but also clinically related disorders such as LBD and Parkinson-plus syndromes. These data underline the complex genetic nature of these neurodegenerative diseases linking them together in spectrum disorders. As only few studies have included patients with PD-related disorders in their mutation analyses, the exact contribution of PD genes to the etiology of other neurodegenerative diseases remains unclear. Even for PD itself, this assessment is not straightforward as most mutation reports present fragmented data on only one or few PD genes. Because more recent data support that dosage plays a major role in PD pathogenesis (e.g., higher frequency of multiplications for *SNCA* than missense mutations and significant contribution of dosage effects in recessive genes), a lot of attention is drawn to gene expression regulation. Noncoding sequence variants in the promoter and UTR regions of PD genes are all potential disease associated variants. Indeed, variants in the *SNCA* promoter and UTR regions (both 5′ and 3′) were reported to be associated with increased risk for PD [Brighina et al., 2008; Farrer et al., 2001b; Hadjigeorgiou et al., 2006; Izumi et al., 2001; Kay et al., 2008; Maraganore et al., 2006; Mizuta et al., 2006; Mueller et al., 2005; Myhre et al., 2008b; Pals et al., 2004b; Parsian et al., 2007; Ross et al., 2007; Tan et al., 2004; Winkler et al., 2007]. Moreover, recent genome-wide association studies (GWAS) in PD patient and control groups confirmed the *SNCA* region as a major player in PD susceptibility [Pankratz et al., 2009; Satake et al., 2009; Simon-Sanchez et al., 2009]. Also, variants located upstream of *LRRK2* were identified to be associated with increased risk for PD, suggesting that variants causing transcriptional upregulation of *LRRK2* might be part of PD etiology [Satake et al., 2009; Simon-Sanchez et al., 2009]. Given that not all is known about the cell's transcription and translation mechanisms, variants detected in previously unexplored genomic regions might turn out to represent a novel group of pathogenic variations. It is clear that the research field should keep an open mind when performing mutation analyses and interpreting its results as exemplified by the variants in the promoter and UTR regions that were previously overlooked. Unfortunately, it is difficult to assess the pathogenic nature of these new subtypes of genetic variants without relevant functional assays. This concern already exists for several other groups of mutations in known PD genes. For example, we do not have unambiguous information on the actual involvement of *PARK2* heterozygotes in PD pathogenicity or on the implications of mutations in *LRRK2* regions with less known functionality. Even the current functional analyses of putative pathogenicity of *LRRK2* mutations might be misleading because no physiological substrate has yet been identified. These concerns signify the urgent need of the molecular genetics field to invest more time and efforts in the development of relevant functional assays.

For the novel PDmutDB database to be usable in the broader research field, data on other genes associated with PD will be added in the near future. Functional data will also be included in the database when these data become available. This way we strive to develop a valuable, easy accessible, and up-to-date instrument for future research or diagnostic purposes.

In conclusion, it is clear that our knowledge on underlying genetics of PD gathered in the last 2 decades has provided researchers with incredible amounts of information on the different biological pathways involved in the pathogenesis of PD. There are still a large number of unanswered questions residing among the few solved mysteries, which will need further attention to fully understand PD in all its facets.
