# Supplementary material for: Genetic Etiology of Parkinson Disease Associated with Mutations in the SNCA, PARK2, PINK1, PARK7, and LRRK2 Genes: A Mutation Update
Source: Hum Mutat. 2010 May 18;31(7):763–80. doi: 10.1002/humu.21277 (PMC3056147; doi:10.1002/humu.21277)
Supplement: Supplementary file 1 [file humu0031-0763-SD1.pdf]

Supp. Table S1-1. *SNCA* Classic mutations [(a)]

Region	NG Numbering	cDNA Numbering	Protein Numbering	dbSNP number	Reference
(a) Missense, nonsense and frameshift mutations					
EX2	g.07717G>C	c.88G>C	p.A30P	-	[Kruger et al., 1998]
EX3	g.15127G>A	c.136G>A	p.E46K	-	[Zarranz et al., 2004]
EX3	g.15148G>A	c.157G>A	p.A53T	-	[Polymeropoulos et al., 1997]
(b) Splice, silent and UTR variants					
Region	NG Numbering	cDNA Numbering	Alias	dbSNP number	Reference
IVS2	g.07759A>C	-	IVS2+9A>C†	-	[Nuytemans et al., 2009]
EX3	g.15138G>A	c.193G>A	p.V49	-	[Nuytemans et al., 2009]
EX6	g.116654C>T	c.454C>T	p.Y136	-	[Nuytemans et al., 2009]
3'UTR	g.117170C>T	c.*501C>T	Ex6+534C>T	rs17016074	[Mueller et al., 2005]
3'UTR	*g.117562C>T*	*c.*893C>T*	*Ex6+926C>T*	*rs356165*	[Pals et al., 2004a]

Supp. Table S1-2. *SNCA* Copy number variations [(a)]

Region	cDNA Numbering	Alias	Mutation Description	Reference
Complete gene	c.(?_-70)_(*2533_?)[3]	tripSNCA	Chromosome 4q genomic triplication of unknown size containing *SNCA* and including *HERC5*, *PIGY*, *HERC3*, *NAPIL5*, *FAM13A0S*, *AX748242*, *FAM13A1*, *KIAA0914*, *TIGD2*, *AK026379*, *GPRIN3*	[Singleton et al., 2003]
Complete gene	c.(?_-70)_(*2533_?)[3]	tripSNCA	Chromosome 4q genomic triplication of unknown size containing SNCA and including *CR05611*, *AK123890*, *MMRN1*, *MGC48628* and *KIAA1680*	[Farrer et al., 2004]
Complete gene	c.(?_-70)_(*2533_?)[3]	tripSNCA	Chromosome 4q genomic triplication of unknown size containing SNCA and including *AK026379*, *GPRIN3*, *CR05611*, *AK123890*, *MMRN1*, *MGC48628*, *KIAA1680* and *TMSL3*	[Ibanez et al., 2009]
Complete gene	c.(?_-70)_(*2533_?)dup	dupSNCA	Chromosome 4q genomic duplication of unknown size containing SNCA and including *CR05611*, *AK123890*, *MMRN1*, *MGC48628* and *KIAA1680*	[Fuchs et al., 2007]
Complete gene	c.(?_-70)_(*2533_?)dup	dupSNCA	Chromosome 4q genomic duplication of unknown size containing SNCA and including at least *FAM13A1*, *KIAA0914*, *TIGD2*, *AK026379*, *GPRIN3* and *CR05611*	[Chartier-Harlin et al., 2004]
Complete gene	c.(?_-70)_(*2533_?)dup	dupSNCA	Chromosome 4q genomic duplication of unknown size containing SNCA and including at least *CR05611* and *AK123890*	[Ibanez et al., 2004]
Complete gene	c.(?_-70)_(*2533_?)dup	dupSNCA	Chromosome 4q genomic duplication of unknown size containing SNCA and including at least *CR05611*, *AK123890* and *MMRN1*	[Ibanez et al., 2004]
Complete gene	c.(?_-70)_(*2533_?)dup	dupSNCA	Chromosome 4q genomic duplication of unknown size containing SNCA and including *CR05611*, *AK123890* and *MMRN1*	[Nishioka et al., 2006]
Complete gene	c.(?_-70)_(*2533_?)dup	dupSNCA	Chromosome 4q genomic duplication of unknown size containing SNCA and including at least *CR05611*, *AK123890*, *MMRN1*, *MGC48628* and *KIAA1680*	[Nishioka et al., 2006]
Complete gene	c.(?_-70)_(*2533_?)dup	dupSNCA	Chromosome 4q genomic duplication of unknown size containing at least *SNCA*	[Ahn et al., 2008; Nuytemans et al., 2009]
Complete gene	c.(?_-70)_(*2533_?)dup	dupSNCA	Chromosome 4q genomic duplication of unknown size containing *SNCA* and including 32 more known or predicted genes	[Ikeuchi et al., 2008]
Complete gene	c.(?_-70)_(*2533_?)dup	dupSNCA	Chromosome 4q genomic duplication of unknown size containing *SNCA* and including 44 more known or predicted genes	[Ibanez et al., 2009]
Complete gene	c.(?_-70)_(*2533_?)dup	dupSNCA	Chromosome 4q genomic duplication of unknown size containing *SNCA* and including 34 more known or predicted genes	[Ibanez et al., 2009]

Region	cDNA Numbering	Alias	Mutation Description	Reference
Complete gene	c.(?_-70)_(*2533_?)dup	dupSNCA	Chromosome 4q genomic duplication of unknown size containing *SNCA* and including *CR05611*, *AK123890*, *MMRN1*, *MGC48628* and *KIAA1680*	[Ibanez et al., 2009]
Complete gene	c.(?_-70)_(*2533_?)dup	dupSNCA	Chromosome 4q genomic duplication of unknown size containing *SNCA* and including *CR05611* and *AK123890*	[Ibanez et al., 2009]

For all references please visit the Parkinson Disease mutation database PDmutDB (http://www.molgen.ua.ac.be/PDmutDB).
[a] NG numbering: NG_011851.1; cDNA numbering: NM_001146055.1; protein numbering: NP_000336.1. Nucleotide numbering reflects cDNA numbering with +1 corresponding to the A of the ATG translation initiation codon in the reference sequence, according to journal guidelines (www.hgvs.org/mutnomen). The initiation codon is codon 1.
† Also observed in control individuals

Supp. Table S2. *LRRK2* Classic mutations [(a)]

(a) Missense, nonsense and frameshift mutations

Region	NG numbering	cDNA Numbering	Protein Numbering	dbSNP number	Reference
EX1	g.5149G>A	c.28G>A	p.E10K	-	[Nichols et al., 2007]
EX2	g.5548C>T	c.155C>T	p.S52F§	rs72546335	[Lesage et al., 2009]
EX4	*g.15624T>C*	*c.356T>C*	*p.L119P*	*rs33995463*	[Zimprich et al., 2004a]
EX6	g.20533C>T	c.632C>T	p.A211V†	-	[Xiromerisiou et al., 2007]
EX6	g.20584G>C	c.647G>C	p.C228S*	rs56108242	[Paisan-Ruiz et al., 2008]
EX9	g.31263G>A	c.1000G>A	p.E334K	-	[Nichols et al., 2007]
EX9	g.31351A>G	c.1088A>G	p.N363S	rs72546336	[Lesage et al., 2009]
EX9	g.31359G>A	c.1096G>A	p.V366M§	-	[Nuytemans et al., 2009]
EX11	*g.32974C>T*	*c.1256C>T*	*p.A419V*	*rs34594498*	[Di Fonzo et al., 2006b]
EX13	g.39568G>A	c.1517G>A	p.R506Q	-	[Nuytemans et al., 2009]
EX14	g.43865A>G	c.1630A>G	p.K544E	-	[Xiromerisiou et al., 2007]
EX14	*g.43888C>G*	*c.1653C>G*	*p.N551K*	*rs7308720*	[Mata et al., 2005b]
EX18	g.58157C>T	c.2147C>T	p.A716V*	-	[Paisan-Ruiz et al., 2008]
EX18	g.58144A>G	c.2134A>G	p.M712V	-	[Paisan-Ruiz et al., 2008]
EX18	*g.58177A>G*	*c.2167A>G*	*p.I723V*	*rs10878307*	[Mata et al., 2005b]
EX19	*g.63887C>T*	*c.2264C>T*	*p.P755L*	*rs34410987*	[Di Fonzo et al., 2006b]
EX19	g.64001G>T	c.2378G>T	p.R793M†	rs35173587	[Zimprich et al., 2004a]
EX19	g.64051A>G	c.2428A>G	p.I810V	rs72546337	[Lesage et al., 2009]
EX20	g.67451A>G	c.2611A>G	p.K871E*	-	[Paisan-Ruiz et al., 2008]
EX21	g.73614G>x	c.2769G>x	p.Q923H	rs58559150	[Camargos et al., 2009]
EX21	g.73634A>G	c.2789A>G	p.Q930R	-	[Berg et al., 2005]
EX25	g.75456G>A	c.2918G>A	p.S973N	-	[Haubenberger et al., 2007]
EX23	g.75556A>G	c.3018A>G	p.I1006M	-	[Nuytemans et al., 2009]
EX24	g.78336G>A	c.3200G>A	p.R1067Q†	-	[Skipper et al., 2005]
EX24	g.78423C>G	c.3287C>G	p.S1096C	-	[Berg et al., 2005]
EX24	g.78469G>T	c.3333G>T	p.Q1111H	-	[Nichols et al., 2007]
EX25	g.79115A>G	c.3364A>G	p.I1122V	rs34805604	[Zimprich et al., 2004a]
EX25	*g.79202G>A*	*c.3451G>A*	*p.A1151T*	-	[Schlitter et al., 2006]
EX25	g.79245T>C	c.3494T>C	p.L1165P	-	[Chen-Plotkin et al., 2008]
EX26	g.82856A>G	c.3574A>G	p.I1192V	-	[Nichols et al., 2007]
EX27	g.83994A>G	c.3647A>G	p.H1216R	rs72546324	[Lesage et al., 2009]
EX27	g.84030G>C	c.3683G>C	p.S1228T	rs60185966	[Berg et al., 2005]
EX28	g.85781C>G	c.3784C>G	p.P1262A*,§	rs4640000	[Paisan-Ruiz et al., 2008]

EX29	g.88471G>A	c.3974G>A	p.R1325Q	rs72546338	[Nuytemans et al., 2008]
EX29	g.88608A>G	c.4111A>G	p.I1371V	rs17466213	[Paisan-Ruiz et al., 2005]
EX30	*g.89099G>A*	*c.4193G>A*	*p.R1398H*	*rs7133914*	[Paisan-Ruiz et al., 2005]
EX30	g.89135C>T	c.4229C>T	p.T1410M	rs72546327	[Lesage et al., 2009]
EX30	g.89164G>A	c.4258GA	p.D1420N	-	[Gao et al., 2009]
EX31	g.90424C>G	c.4321C>G	p.R1441G	-	[Paisan-Ruiz et al., 2004]
EX31	g.90424C>T	c.4321C>T	p.R1441C	rs33939927	[Zimprich et al., 2004a]
EX31	g.90425G>A	c.4322G>A	p.R1441H	rs34995376	[Mata et al., 2005b]
EX31	g.90427G>C	c.4324G>C	p.A1442P	-	[Huang et al., 2007]
EX31	g.90440C>T	c.4337C>T	p.P1446L	-	[Zabetian et al., 2009]
EX31	g.90451G>A	c.4348G>A	p.V1450I	-	[Zabetian et al., 2009]
EX31	g.90505A>G	c.4402A>G	p.K1468E	-	[Nuytemans et al., 2008]
EX31	g.90551G>A	c.4448G>A	p.R1483Q	-	[Nuytemans et al., 2008]
EX32	*g.93966G>A*	*c.4541G>A*	*p.R1514Q*	*rs35507033*	[Zimprich et al., 2004a]
EX32	*g.94049C>T*	*c.4624C>T*	*p.P1542S*	*rs33958906*	[Mata et al., 2005b]
EX34	*g.99988T>C*	*c.4838T>C*	*p.V1613A*	-	[Pchelina et al., 2008]
EX34	*g.100036G>C*	*c.4883G>C*	*p.R1628P*	*rs33949390*	[Mata et al., 2005b]
EX34	*g.100087T>C*	*c.4937T>C*	*p.M1646T*	*rs35303786*	[Paisan-Ruiz et al., 2004]
EX34	*g.100089T>A*	*c.4939T>A*	*p.S1647T*	*rs11564148*	[Mata et al., 2005b]
EX35	g.101104A>T	c.5096A>G	p.Y1699C	rs35801418	[Paisan-Ruiz et al., 2004]
EX36	g.102037G>A	c.5183G>A	p.R1728H	-	[Paisan-Ruiz et al., 2008]
EX36	g.102037G>T	c.5183G>T	p.R1728L	-	[Paisan-Ruiz et al., 2008]
EX37	g.102376G>T	c.5385G>T	p.L1795F	-	[Nichols et al., 2007]
EX38	g.103245A>G	c.5605A>G	p.M1869V	-	[Di Fonzo et al., 2006b]
EX38	g.103246T>C	c.5606T>C	p.M1869T†	rs35602796	[Farrer et al., 2005]
EX38	g.103250G>T	c.5610G>T	p.L1870F*	-	[Paisan-Ruiz et al., 2008]
EX38	g.103260G>T	c.5620G>T	p.E1874X	-	[Di Fonzo et al., 2006b]
EX40	g.115021G>A	c.5822G>A	p.R1941H	-	[Khan et al., 2005b]
EX41	g.120351T>C	c.6016T>C	p.Y2006H	-	[Lesage et al., 2007a]
EX41	g.120370T>C	c.6035T>C	p.I2012T	rs34015634	[Tomiyama et al., 2006]
EX41	g.120390G>A	c.6055G>A	p.G2019S†	rs34637584	[Di Fonzo et al., 2005]
EX41	g.120394T>C	c.6059T>C	p.I2020T	rs35870237	[Zimprich et al., 2004a]
EX41	g.120426A>T	c.6091A>T	p.T2031S	-	[Lesage et al., 2007a]
EX42	*g.126874A>G*	*c.6241A>G*	*p.N2081D*	*rs33995883*	[Zimprich et al., 2004a]
EX44	g.131569C>T	c.6422C>T	p.T2141M	-	[Paisan-Ruiz et al., 2008]
EX44	g.131575G>A	c.6428G>A	p.R2143H	-	[Paisan-Ruiz et al., 2008]

EX44	g.131713A>G	c.6566A>G	p.Y2189C	rs35658131	[Nuytemans et al., 2008]
EX48	g.143430C>T	c.7067C>T	p.T2356I	-	[Khan et al., 2005b]
EX48	*g.143516G>A*	*c.7153G>A*	*p.G2385R*	*rs34778348*	[Mata et al., 2005b]
EX48	g.143531G>A	c.7168G>A	p.V2390M	-	[Clarimon et al., 2008]
EX49	g.144833G>A	c.7183G>A	p.E2395K*,§	-	[Paisan-Ruiz et al., 2008]
EX49	*g.144840T>C*	*c.7190T>C*	*p.M2397T*	*rs3761863*	[Paisan-Ruiz et al., 2005]
EX50	g.147002T>A	c.7397T>A	p.L2466H	-	[Paisan-Ruiz et al., 2008]
EX51	g.147639delC	c.7468delC	p.Q2490NfsX3†	-	[Nuytemans et al., 2009]

(b) Splice, silent and UTR variants

Region	NG numbering	cDNA Numbering	Alias	dbSNP number	Reference
5'UTR	g.5078G>T	c.-44G>T	c.-44G>T	-	[Paisan-Ruiz et al., 2008]
5'UTR	g.5092G>C	c.-30G>C	c.-30G>C	rs72546314	[Lesage et al., 2009]
EX5	*g.17979T>C*	*c.457T>C*	*p.L153*	*rs10878245*	[Mata et al., 2005b]
EX5	*g.18068A>G*	*c.546A>G*	*p.K182*	*rs35517158*	[Mata et al., 2005b]
EX7	*g.23658T>C*	*c.825T>C*	*p.H275*	-	[Paisan-Ruiz et al., 2008]
EX8	g.29844C>T	c.867C>T	p.N289	rs17490713	[Paisan-Ruiz et al., 2008]
EX8	g.29913G>T	c.936G>T	p.A312	rs41286466	[Nuytemans et al., 2009]
IVS9	*g.31445G>A*	-	*IVS9-10C>A*	*rs7955902*	[Di Fonzo et al., 2006a]
IVS10	g.32896A>G	-	IVS10-4A>G*	rs7135747	[Paisan-Ruiz et al., 2008]
EX12	*g.37332T>C*	*c.1383T>C*	*p.S461*	*rs35847451*	[Mata et al., 2005b]
EX13	*g.39515A>T*	*c.1464A>T*	*p.L488*	-	[Nichols et al., 2007]
IVS16	g.57864delT	-	IVS16-14delT*	-	[Paisan-Ruiz et al., 2008]
EX17	g.57958A>C	c.2022A>C	p.V674	rs72546319	[Lesage et al., 2009]
EX18	g.58131C>T	c.2121C>T	p.Y707*	-	[Paisan-Ruiz et al., 2008]
EX19	*g.64106T>C*	*c.2481T>C*	*p.S827*	-	[Nichols et al., 2007]
IVS19	g.64128_64131delGTAA	-	IVS19+5_8delGTAA	-	[Johnson et al., 2007]
IVS19	*g.67332_67333insT*	-	*IVS19-9_8insT*	*rs36220740*	[Di Fonzo et al., 2006a]
EX22	*g.74883T>C*	*c.2857T>C*	*p.L953*	*rs7966550*	[Paisan-Ruiz et al., 2005]
EX24	*g.78478A>G*	*c.3342A>G*	*p.L1114§*	*rs35808389*	[Zimprich et al., 2004a]
IVS25	g.82771delT	-	IVS25-8delT†	-	[Paisan-Ruiz et al., 2008]
IVS27	g.85764C>T	-	IVS27-9C>T	-	[Paisan-Ruiz et al., 2008]
EX30	*g.89175G>A*	*c.4269G>A*	*p.K1423*	*rs11175964*	[Mata et al., 2005b]
EX30	g.89196C>T	c.4290C>T	p.A1430	-	[Paisan-Ruiz et al., 2008]
IVS30	*g.89235delT*	-	*IVS30+12delT†*	-	[Di Fonzo et al., 2006a]
IVS30	g.90415C>T	-	IVS30-6C>T	rs56082834	[Nuytemans et al., 2009]
EX31	g.90426C>T	c.4323C>T	p.R1441§	-	[Gaig et al., 2008]

IVS31	g.90642A>G	-	IVS31+3A>G	rs41286476	[Zabetian et al., 2005]
IVS32	g.94177G>A	-	IVS32+14G>A	rs72546328	[Lesage et al., 2009]
IVS33	g.95296T>A	-	IVS33+6T>A	-	[Skipper et al., 2005]
EX34	*g.100022C>A*	*c.4872C>A*	*p.G1624*	*rs1427263*	[Paisan-Ruiz et al., 2005]
EX34	*g.100061A>G*	*c.4911A>G*	*p.K1637*	*rs11176013*	[Mata et al., 2005b]
EX37	*g.102448T>C*	*c.5457T>C*	*p.G1819*	*rs10878371*	[Mata et al., 2005b]
IVS37	g.103141A>G	-	IVS37-9A>G†	rs41286460	[Di Fonzo et al., 2006a]
IVS38	g.103303C>T	-	IVS38+7C>T	rs36212069	[Paisan-Ruiz et al., 2008]
EX41	*g.120348C>T*	*c.6013C>T*	*p.L2005**	-	[Orr-Urtreger et al., 2007]
EX41	*g.120389C>T*	*c.6054C>T*	*p.Y2018**	-	[Orr-Urtreger et al., 2007]
EX43	*g.128442G>A*	*c.6324G>A*	*p.E2108*	*rs10878405*	[Mata et al., 2005b]
EX44	*g.131657C>A*	*c.6510C>A*	*p.G2170*	*rs34869625*	[Mata et al., 2005b]
IVS46	g.139236T>A	-	IVS46-14T>A	rs72546333	[Lesage et al., 2009]
IVS46	g.139242delT	-	IVS46-8delT	rs72546334	[Lesage et al., 2009]
IVS47	g.143383delT	-	IVS47-9delT	rs11317573	[Di Fonzo et al., 2006a]
EX48	*g.143518A>G*	*c.7155A>G*	*p.G2385*	*rs33962975*	[Mata et al., 2005b]
EX49	g.144946C>G	c.7296C>G	p.G2432*	-	[Paisan-Ruiz et al., 2008]

For all references please visit the Parkinson Disease mutation database PDmutDB (http://www.molgen.ua.ac.be/PDmutDB).
[(a)] NG numbering: NG_011709.1; cDNA numbering: NM_198578.2; protein numbering: NP_940980.3. Nucleotide numbering reflects cDNA numbering with +1 corresponding to the A of the ATG translation initiation codon in the reference sequence, according to journal guidelines (www.hgvs.org/mutnomen). The initiation codon is codon 1.
* Only observed in control individuals;
† Also observed in control individuals;
§ In proximity to exon/intron boundary;
Italic: known polymorphism

Supp. Table S3-1. *PARK2* Classic mutations [(a)]

(a) Missense, nonsense and frameshift mutations

Region	NG numbering	cDNA Numbering	Protein Numbering	Alias	dbSNP number	Reference
EX1	g.0005135A>T	c.1A>T	p.0?	Met1Leu	-	[Rawal et al., 2003]
EX	g.0289335G>A	c. 13G>A	p.V5I	Val5Ile	-	[Camargos et al., 2009]
EX2	g.0289351G>A	c.29G>A	p.S10N	Ser10Asn	-	[Nuytemans et al., 2009]
EX2	g.0289356G>C	c.34G>C	p.G12R	Gly12Arg	-	[Mellick et al., 2009]
EX2	g.0289365G>A	c.43G>A	p.V15M	Val15Met	-	[Munoz et al., 2002]
EX2	g.0289374G>A	c.52G>A	p.D18N	Asp18Asn	-	[Annesi et al., 2007]
EX2	g.0289414C>A	c.92C>A	p.A31D	Ala31Asp	-	[Chaudhary et al., 2006]
EX2	g.0289417A>C	c.95A>C	p.K32T	Lys32Thr	-	[Tarantino et al., 2007]
EX2	g.0289419C>T	c.97C>T	p.R33X	Arg33Stop	-	[Maruyama et al., 2000]
EX2	g.0289420G>A	c.98G>A	p.R33Q	Arg33Gln	-	[Hertz et al., 2006]
EX2	g.0289423delA	c.101delA	p.Q34RfsX10	Gln34fs	-	[Nisipeanu et al., 2001]
EX2	g.0289423_0289424delAG	c.101_102delAG	p.Q34RfsX5	Gln34fs	rs55777503	[Abbas et al., 1999]
EX2	*g.0289423A>G*	*c.101A>G*	*p.Q34R*	*Gln34Arg*	-	[Biswas et al., 2006]
EX2	g.0289432C>T	c.110C>T	p.P37L	Pro37Leu	-	[Kann et al., 2002]
EX2	g.0289440C>T	c.118C>T	p.Q40X	Gln40Stop	-	[Choi et al., 2008]
EX2	g.0289446C>T	c.124C>T	p.R42C	Arg42Cys	-	[Biswas et al., 2006]
EX2	g.0289447G>A	c.125G>A	p.R42H	Arg42His	-	[Madegowda et al., 2005]
EX2	g.0289447G>C	c.125G>C	p.R42P	Arg42Pro	-	[Terreni et al., 2001]
EX2	g.0289458G>A	c.136G>A	p.A46T	Ala46Thr	-	[Okubadejo et al., 2008]
EX2	g.0289458G>C	c.136G>C	p.A46P	Ala46Pro	-	[Xu et al., 2002]
EX2	g.0289477delA	c.155delA	p.N52MfsX29	Asn52fs	-	[Lucking et al., 2000]
EX2	g.0289478_0289479insT	c.156_157insT	p.D53X	Asp53Stop	-	[Aguiar et al., 2008]
EX2	g.0289482T>A	c.160T>A	p.W54R	Trp54Arg	-	[Camargos et al., 2009]
EX2	g.0289486C>T	c.164C>T	p.T55I	Thr55Ile	-	[Rocca et al., 2007]
EX2	g.0289489T>A	c.167T>A	p.V56E	Val56Glu	-	[Hoenicka et al., 2002]
EX3	g.0470086_0470087insGT	c.220_221insGT	p.W74CfsX8	Trp74fs	-	[Abbas et al., 1999]
EX3	g.0470101G>T	c.235G>T	p.E79X	Glu79Stop	-	[Foroud et al., 2003]
EX3	g.0470111C>A	c.245C>A	p.A82E†	Ala82Glu	rs55774500	[Hedrich et al., 2001]
EX3	g.0470122G>A	c.256G>A	p.D86N	Asp86Asn	-	[Ciro, I et al., 2007]
EX3	*g.0470166G>C*	*c.300G>C*	*p.Q100H*	*Gln100His*	-	[Chen et al., 2003]
EX3	g.0470176C>T	c.310C>T	p.R104W	Arg104Trp	-	[Varrone et al., 2004]
EX3	g.0470203_0470242del40	c.337_376del40	p.P113TfsX51	Pro113fs / ex3 Δ40bp	-	[Farrer et al., 2001a]
EX3	g.0470263_0470265delCCA	c.397_399delCCA	p.P133del	Pro133del	-	[Hedrich et al., 2004b]

EX3	g.0531566A>G	c.428A>G	p.Y143C	Tyr134Cys	-	[Biswas et al., 2006]
EX4	g.0531572G>A	c.434G>A	p.S145N	Ser145Asn	-	[Varrone et al., 2004]
EX4	g.0531596C>G	c.458V>G	p.P153R†	Pro153Arg	rs55654276	[Wang et al., 2008]
EX4	g.0531621A>T	c.483A>T	p.K161N	Lys161Asn	-	[Abbas et al., 1999]
EX4	g.0531635G>A	c.497G>A	p.C166T	Cys166Tyr	-	[Brooks et al., 2009]
EX4	*g.051638G>A*	*c.500G>A*	*p.S167N*	*Ser167Asn*	*rs1801474*	[Hattori et al., 1998a]
EX4	g.0531649C>T	c.511C>T	p.Q171X	Gln171Stop	-	[Brooks et al., 2009]
EX4	g.0531656C>T	c.518C>T	p.T173M*	Thr173Met	-	[Kay et al., 2007]
EX5	g.0678630delG	c.536delG	p.G179VXfs9	Gly179fs	-	[Hattori et al., 1998a]
EX5	*g.0678668A>C*	*c.574A>C*	*p.M192L*	*Met192Leu*	*rs9456735*	[Hedrich et al., 2002]
EX5	g.0678668A>G	c.574A>G	p.M192V	Met192Val	-	[Dachsel et al., 2006]
EX5	g.0678694C>G	c.600C>G	p.H200Q	His200Gln	rs72480421	[Bardien et al., 2009]
EX6	g.0759399A>G	c.632A>G	p.K211R	Lys211Arg	-	[Periquet et al., 2001]
EX6	g.0759400A>T	c.633A>T	p.K211N	Lys211Asn	-	[Lucking et al., 2000]
EX6	g.0759401delT	c.634delT	p.C212VfsX13	Cys212fs	-	[Sironi et al., 2008]
EX6	g.0759401T>G	c.634T>G	p.C212G	Cys212Gly	-	[Shyu et al., 2005]
EX6	g.0759402G>A	c.635G>A	p.C212Y	Cys212Tyr	-	[Pineda-Trujillo et al., 2001]
EX6	g.0759412C>A	c.645C>A	p.H215Q	His215Gln	-	[Pigullo et al., 2004]
EX6	g.0759455G>A	c.688G>A	p.A230T	Ala230Thr	-	[Sironi et al., 2008]
EX6	g.0759468G>A	c.701G>A	p.R234Q	Arg234Gln	-	[Hertz et al., 2006]
EX6	g.0759481C>G	c.714G>A	p.C238W	Cys238Trp	-	[Sironi et al., 2008]
EX6	g.0759486C>G	c.719C>G	p.T240R	Thr240Arg	-	[Hattori et al., 1998b]
EX6	g.0759486C>T	c.719C>T	p.T240M	Thr240Met	-	[Periquet et al., 2003]
EX6	g.0759494G>A	c.727G>A	p.D243N†	Asp243Asn	-	[Romito et al., 2005]
EX6	g.0759497G>A	c.730G>A	p.V244I*,§	Val244Ile	-	[Kay et al., 2007]
EX7	g.0946918G>A	c.758G>A	p.C253Y	Cys253Tyr	-	[Sironi et al., 2008]
EX7	g.0946918G>T	c.758G>T	p.C253F	Cys253Phe	-	[Guo et al., 2008]
EX7	g.0946919C>G	c.759C>G	p.C253W	Cys253Trp	-	[Oliveira et al., 2003]
EX7	g.0946926C>T	c.766C>T	p.R256C	Arg256Cys	-	[Abbas et al., 1999]
EX7	g.0946932G>A	c.772G>A	p.V258M	Val258Met	-	[Shyu et al., 2005]
EX7	g.0946954A>G	c.794A>G	p.H265R	His265Arg	-	[Nuytemans et al., 2009]
EX7	g.0946959T>C	c.799T>C	p.Y267H	Tyr267His	-	[Lee et al., 2009]
EX7	g.0946964T>A	c.804T>A	p.C268X	Cys268Stop	-	[Lucking et al., 2000]
EX7	*g.0946973A>T*	*c.813A>T*	*p.R271S*	*Arg271Ser*	-	[Chen et al., 2003]
EX7	g.0946974C>A	c.814C>A	p.L272I	Leu272Ile	-	[Li et al., 2005a]
EX7	g.0946978A>G	c.818A>G	p.N273S†	Asn273Ser	-	[Lesage et al., 2008]

EX7	g.0946983C>T	c.823C>T	p.R275W†	Arg275Trp	rs34424986	[Abbas et al., 1999]
EX7	g.0946998G>A	c.838G>A	p.D280N	Asp280Asn	rs72480422	[Lucking et al., 2000]
EX7	g.0947008T>C	c.848T>C	p.L283P	Leu283Pro	rs56754308	[Macedo et al., 2009]
EX7	g.0947010G>C	c.850G>C	p.G284R	Gly284Arg	-	[Wu et al., 2005]
EX7	g.0947025T>G	c.865T>G	p.C289G§	Cys289Gly	rs55961220	[Lucking et al., 2000]
EX7	g.0947031delG	c.871delG	p.A291LfsX7	Ala291fs	-	[Munoz et al., 2000]
EX8	g.1163407A>C	c.892A>C	p.I298L	Ile298Leu	-	[Wang et al., 2008]
EX8	g.1163408T>G	c.893T>G	p.I298S	Ile298Ser	-	[Lesage et al., 2008]
EX8	g.1163445G>C	c.930G>C	p.E310D	Glu310Asp	rs72480423	[Bardien et al., 2009]
EX8	g.1163446C>T	c.931C>T	p.Q311X	Gln311Stop	-	[Hattori et al., 1998b]
EX8	g.1163448G>T	c.933G>T	p.Q311H§	Gln311His	-	[Shyu et al., 2005]
EX9	g.1183834_1183839del6	c.968_973delGTGTCC	p.C323_V324del	Cys323fs	-	[Guo et al., 2008]
EX9	g.1183837delT	c.971delT	p.V324AfsX111	Val324fs	-	[Klein et al., 2000]
EX9	g.1183849G>A	c.983G>A	p.G328E	Gly328Glu	-	[Lucking et al., 2000]
EX9	g.1183866C>T	c.1000C>T	p.R334C†	Arg334Cys	-	[Lucking et al., 2000]
EX9	g.1183867G>A	c.1001G>A	p.R334H	Arg334His	-	[Okubadejo et al., 2008]
EX9	*g.1183881G>T*	*c.1015G>T*	*p.A339S*	*Ala339Ser*	-	[Chen et al., 2003]
EX9	g.1183907_1183908delGA	c.1041_1042delGA	p.R348EfsX21	Arg348fs	-	[Lucking et al., 2000]
EX9	g.1183912_1183913delAA	c.1046_1047delAA	p.K349SfsX20	Lys349fs	-	[Hedrich et al., 2001]
EX9	g.1183917A>C	c.1051A>C	p.T351P	Thr351Pro	-	[Kann et al., 2002]
EX9	g.1183942G>A	c.1076G>A	p.G359D†	Gly359Asp	-	[Madegowda et al., 2005]
EX10	*g.1345938C>T*	*c.1096C>T*	*p.R366W*	*Arg366Trp*	*rs56092260*	[Wang et al., 1999]
EX10	g.1345939G>A	c.1097G>A	p.R366Q	Arg366Gln	-	[Wang et al., 2008]
EX10	*g.1345980G>C*	*c.1138G>C*	*p.V380L*	*Val380Leu*	*rs1801582*	[Abbas et al., 1999]
EX11	g.1372605delGA	c.1175_1176delGA	p.R392SfsX3	Arg392fs	-	[Alvarez et al., 2001]
EX11	*g.1372610G>A*	*c.1180G>A*	*p.D394N*	*Asp394Asn*	*rs1801334*	[Abbas et al., 1999]
EX11	g.1372613G>T	c.1183G>T	p.E395X	Glu395Stop	-	[Bras et al., 2008]
EX11	g.1372616A>G	c.1186A>G	p.R396G	Arg396Gly	-	[Wu et al., 2005]
EX11	g.1372622G>A	c.1192G>A	p.A398T	Ala398Thr	-	[Periquet et al., 2003]
EX11	g.1372634C>T	c.1204C>T	p.R402C†	Arg402Cys	rs55830907	[Poorkaj et al., 2004]
EX11	g.1372635G>A	c.1205G>A	p.R402H	Arg402His	-	[Sun et al., 2006]
EX11	g.1372655G>T	c.1225G>T	p.E409X	Glu409Stop	-	[Sironi et al., 2008]
EX11	g.1372674C>A	c.1244C>A	p.T415N†	Thr415Asn	-	[Abbas et al., 1999]
EX11	g.1372682T>C	c.1252T>C	p.C418R	Cys418Arg	-	[Bertoli-Avella et al., 2005]
EX11	g.1372691_1372692insA	c.1283_1284insA	p.N428KfsX141	Asn428fs	-	[Rawal et al., 2003]
EX12	g.1382592G>A	c.1286G>A	p.G429E§	Gly429Glu	-	[Bruggemann et al., 2009]

EX12	g.1382595G>A	c.1289G>A	p.G430D	Gly430Asp	-	[Lucking et al., 2000]
EX12	g.1382598G>T	c.1292G>T	p.C431F	Cys431Phe	-	[Maruyama et al., 2000]
EX12	g.1382616C>T	c.1310C>T	p.P437L†	Pro437Leu	-	[Hedrich et al., 2002]
EX12	g.1382627T>C	c.1321T>C	p.C441R	Cys441Arg	-	[Shyu et al., 2005]
EX12	g.1382636G>C	c.1330G>C	p.E444Q	Glu444Gln	-	[Madegowda et al., 2005]
EX12	g.1382641G>A	c.1335G>A	p.W445X	Trp445Stop	-	[Rawal et al., 2003]
EX12	g.1382664G>A	c.1358G>A	p.W453X	Trp453Stop	rs34424986	[Abbas et al., 1999]
EX12	g.1382678A>C	c.1372A>C	p.M458L	Met458Leu	-	[Brooks et al., 2009]
EX12	g.1382684_1382685insG	c.1378_1379insG	p.D460GfsX109	Asp460fs	-	[Chan et al., 2008]
EX12	g.1382699G>A	c.1393G>A	p.V465M	Val465Met	-	[Pigullo et al., 2004]

(b) Splice, silent and UTR variants

Region	NG numbering	cDNA Numbering	Protein Numbering	Alias	dbSNP number	Reference
5'UTR	g.0005082C>T	c.-53C>T	p.?*	c.-53C>T	-	[Kay et al., 2007]
5'UTR	g.0005091C>A	c.-44C>A	p.?	c.-44C>A	-	[Kay et al., 2007]
5'UTR	g.0005096G>T	c.-39G>T	p.?	c.-39G>T	-	[Bruggemann et al., 2009]
5'UTR	g.0005114G>T	c.-21G>T	p.?	c.-21G>T	-	[Hedrich et al., 2002]
5'UTR	g.0005129G>C	c.-6G>C	p.?	c.-6G>C	-	[Varrone et al., 2004]
IVS1	g.0005142G>A	-	p.?	IVS1+1G>A	-	[Illarioshkin et al., 2003]
EX2	g.0289370G>A	c.48G>A	p.E16†	Glu16	-	[Okubadejo et al., 2008]
EX3	g.0289373C>x	c.51C>x	p.V17	Val17	-	[Myhre et al., 2008a]
EX3	g.0289433G>A	c.111G>A	p.P37	Pro37	-	[Okubadejo et al., 2008]
EX3	*g.047238C>T*	*c.372C>T*	*p.H124*	*His124*	-	[Chen et al., 2003]
EX4	g.0531660C>T	c.522C>T	p.L174	Leu174	-	[Brooks et al., 2009]
EX4	g.0531669C>A	c.531C>A	p.T177*,§	Thr177	-	[Kay et al., 2007]
IVS4	g.0531682T>C	-	p.?	IVS4+10T>C	-	[Chan et al., 2008]
IVS5	g.0678714T>A	-	p.?	IVS5+2T>A	-	[Scherfler et al., 2004]
EX7	g.0946925A>T	c.765A>T	p.S255†	Ser255	-	[Kay et al., 2007]
EX7	g.0946943A>G	c.783A>G	p.L261	Leu261	rs9456711	[Hedrich et al., 2002]
EX7	g.0946976C>T	c.816C>T	p.L272	Leu272	-	[Chaudhary et al., 2006]
EX7	g.0946997C>T	c.837C>T	p.H279	His279	-	[Pigullo et al., 2004]
IVS7	g.1163384C>G	-	p.?	IVS7-3C>G	-	[Nuytemans et al., 2009]
IVS7	g.1163386G>C	-	p.?	IVS7-1G>C	-	[Rawal et al., 2003]
EX8	g.1163434C>T	c.919C>T	p.L307	Leu307	-	[Sironi et al., 2008]
IVS8	g.1183797C>T	-	p.?	IVS8-3C>T	-	[Pigullo et al., 2004]
EX9	g.1183823T>C	c.957T>C	p.G319	Gly319	-	[Okubadejo et al., 2008]
EX9	g.1183883G>A	c.1017G>A	p.A339	Ala339	-	[Abbas et al., 1999]

IVS9	g.1345925G>A	-	p.?	IVS9-1G>A	-	[Nuytemans et al., 2009]
EX11	g.1372636T>C	c.1206T>C	p.R402	Arg402	-	[Bardien et al., 2009]
IVS11	g.1382589C>G	-	p.?/p.G429EfsX5	IVS11-3C>G	-	[Bertoli-Avella et al., 2005]
3'UTR	g.1382720G>A	c.*16G>A	p.?	*16G>A	rs61730194	[Bardien et al., 2009]
3'UTR	g.1382798A>G	c.*94A>G	p.?†	*94A>G	rs62637702	[Sironi et al., 2008]
3'UTR	g.1382798_1382799delins4	c.*94_*95delinsGCGC	p.?	*94_95delinsGCGC	-	[Sironi et al., 2008]
3'UTR	g.1382807C>T	c.*103C>T	p.?	*103C>T	-	[Bardien et al., 2009]

Supp. Table S3-2. *PARK2* copy number variations (a)

Region	NG Numbering	cDNA Numbering	Protein Numbering	Alias	Reference
Prom+EX1	-	c.(8-?)_oPACRG:(157-?)del	p.0	prom+ex1del (NP)	[Lesage et al., 2007b]
EX1	-	c.(-134-?)_(7+?)del	p.?	ex1del (NP)	[Hedrich et al., 2002]
EX1	-	c.(-134-?)_(7+?)del	p.?	ex1dup (NP)	[Sun et al., 2006]
EX1-4	-	c.(-134-?)_(534+?)del	p.?	ex1-4del (NP)	[Chung et al., 2008]
EX2	-	c.(8-?)_(171+?)del	p.V3EfsX3	ex2del (NP)	[Lucking et al., 2000]
EX2	-	c.(8-?)_(171+?)dup	p.?/p.N58CfsX41†	ex2dup (NP)	[Simon-Sanchez et al., 2008]
EX2	-	c.(8-?)_(171+?)[3]	p.N58CfsX41	ex2trip (NP)	[Lucking et al., 2000]
EX2-3	-	c.(8-?)_(412+?)del	p.V3AfsX329	ex2-3del (NP)	[Lucking et al., 2000]
EX2-3	-	c.(8-?)_(412+?)dup	p.?/p.A138VfsX464	ex2-3dup (NP)	[Nichols et al., 2002]
EX2-4	-	c.(8-?)_(534+?)del	p.V3GfsX7	ex2-4del (NP)	[Maruyama et al., 2000]
EX2-4	-	c.(8-?)_(534+?)dup	p.?/p.G179CfsX41†	ex2-4dup (NP)	[Periquet et al., 2003]
EX2-4	-	c.(8-?)_(534+?)[3]	p.?/p.G179CfsX41	ex2-4trip (NP)	[Mata et al., 2005a]
EX2-5	-	c.(8-?)_(618+?)del	p.V3GfsX5	ex2-5del (NP)	[Kann et al., 2002]
EX2-12	-	c.(8-?)_(3939+?)dup	p.?	ex2-12dup (NP)	[Nuytemans et al., 2009]
EX3	-	c.(172-?)_(412+?)del	p.N58QfsX39	ex3del (NP)	[Hattori et al., 1998a]
EX3	-	c.(172-?)_(412+?)dup	p.?;p.A138EfsX3	ex3dup (NP)	[Lucking et al., 2000]
EX3-4	-	c.(172-?)_(534+?)del	p.N58_Q178del	ex3-4del (NP)	[Hattori et al., 1998a]
EX3-4	-	c.(172-?)_(534+?)dup	p.?/p.N58_Q178dup	ex3-4dup (NP)	[Periquet et al., 2003]
EX3-5	-	c.(172-?)_(618+?)del	p.N58_A206del	ex3-5del (NP)	[Kobayashi et al., 2000]
EX3-6	-	c.(172-?)_(734+?)del	p.N58EfsX29	ex3-6del (NP)	[Lucking et al., 2000]
EX3-7	-	c.(172-?)_(871+?)del	p.A291LfsX7	ex3-7del (NP)	[Kitada et al., 1998]
EX3-9	-	c.(172-?)_(1083+?)del	p.N58_G361del	ex3-9del (NP)	[Lucking et al., 2000]
EX4	g.0484957_0641130del	c.(413-46594)_(534+109486)del156202	p.A138GfsX7	ex4del	[Clarimon et al., 2005]
EX4	g.0529909_0531657del1749	c.(413-1642)_519del1749	p.G139HfsX11	partial ex4del	[Khan et al., 2005a]
EX4	-	c.(413-?)_(534+?)del	p.A138GfsX7	ex4del (NP)	[Kitada et al., 1998]
EX4	-	c.(413-?)_(534+?)dup	p.?/p.G179QfsX39	ex4dup (NP)	[Hedrich et al., 2001]

EX4-5	-	c.(413-?)_(618+?)del	p.G139NfsX18	ex4-5del (NP)	[Kann et al., 2002]
EX4-6	-	c.(413-?)_(734+?)del	p.A138GfsX14	ex4-6del (NP)	[Nichols et al., 2002]
EX4-7	-	c.(413-?)_(871+?)del	p.R140CXfs174	ex4-7del (NP)	[Rawal et al., 2003]
EX5	g.0574670_0702743del12813	c.(535-104015)_(618+24031)del128130	p.G179_A206del	ex5del	[Bayrakli et al., 2007]
EX5	-	c.(535-?)_(618+?)del	p.G179_A206del	ex5del (NP)	[Hattori et al., 1998a]
EX5	-	c.(535-?)_(618+?)dup	p.?/p.G179_A206dup	ex5dup (NP)	[Hedrich et al., 2002]
EX5-6	-	c.(535-?)_(734+?)del	p.G179EfsX29	ex5-6del (NP)	[Lucking et al., 2000]
EX5-7	-	c.(535-?)_(871+?)del	p.G179LfsX7	ex5-7del (NP)	[Leroy et al., 1998a]
EX5-8	-	c.(535-?)_(933+?)dup	p.?/p.G179_Q311dup	ex5-8dup (NP)	[Hertz et al., 2006]
EX5-9	-	c.(535-?)_(1083+?)dup	p.?/p.G179_G361dup	ex5-9dup (NP)	[Brooks et al., 2009]
EX5-12	-	c.(535-?)_(3939+?)del	p.G179_V465del	ex5-12del (NP)	[Moro et al., 2008]
EX6	-	c.(619-?)_(734+?)del	p.F208PfsX28†	ex6del (NP)	[Nichols et al., 2002]
EX6	-	c.(619-?)_(734+?)dup	p.?/p.S246NfsX18	ex6dup (NP)	[Lucking et al., 2000]
EX6-7	-	c.(619-?)_(871+?)del	p.E207LfsX7	ex6-7del (NP)	[Maruyama et al., 2000]
EX6-8	-	c.(619-?)_(933+?)dup	p.?/p.F207_Q311dup	ex6-8dup (NP)	[Sun et al., 2006]
EX7	-	c.(735-?)_(871+?)del	p.R245SfsX9	ex7del (NP)	[Klein et al., 2000]
EX7	-	c.(735-?)_(871?)dup	p.?/p.A291GfsX14†	ex7dup (NP)	[Lucking et al., 2000]
EX7-8	-	c.(735-?)_(933+?)del	p.R245SfsX124	ex7-8del (NP)	[Sun et al., 2006]
EX7-9	-	c.(735-?)_(1083+?)del	p.R245SfsX74	ex7-9del (NP)	[Lucking et al., 2000]
EX8	-	c.(872-?)_(933+?)del	p.A291VfsX35	ex8del (NP)	[Lucking et al., 2000]
EX8	-	c.(872-?)_(933+?)dup	p.?/p.Y312LfsX7	ex8dup (NP)	[Nichols et al., 2002]
EX8-9	-	c.(872-?)_(1083+?)del	p.A291VfsX8	ex8-9del (NP)	[Lucking et al., 1998]
EX8-10	-	c.(872-?)_(1167+?)del	p.A291GfsX6	ex8-10del (NP)	[Bertoli-Avella et al., 2005]
EX8-11	-	c.(872-?)_(1285+?)del	p.A291GfsX38	ex8-11del (NP)	[Bras et al., 2008]
EX9	-	c.(934-?)_(1083+?)dup	p.?/p.Y312_G361dup	ex9dup (NP)	[Kann et al., 2002]
EX10	-	c.(1084-?)_1167+?)del	p.F339AfsX77	ex10del (NP)	[Periquet et al., 2003]
EX10	-	c.(1084-?)_1167+?)dup	p.?/p.A390FfsX105	ex10dup (NP)	[Foroud et al., 2003]
EX10-12	-	c.(1084-?)_(3939+?)del	p.?	ex10-12del (NP)	[Shyu et al., 2005]
EX10-12	-	c.(1084-?)_(3939+?)dup	p.?/p.F362_V466dup	ex10-12dup (NP)	[Macedo et al., 2009]
EX11	-	c.(1168-?)_(1285+?)del	p.A390EfsX6	ex11del (NP)	[Hertz et al., 2006]
EX11	-	c.(1168-?)_(1285+?)dup	p.?/p.G430LfsX4	ex11dup (NP)	[Lucking et al., 2000]
EX12	-	c.(1286-?)_(3939+?)dup	p.?	ex12dup (NP)	[Sun et al., 2006]

For all references please visit the Parkinson Disease mutation database PDmutDB (http://www.molgen.ua.ac.be/PDmutDB).

[(a)] NG numbering: NG_008289.1; cDNA numbering: NM_004562.2; protein numbering: NP_004553.2. Nucleotide numbering reflects cDNA numbering with +1 corresponding to the A of the ATG translation initiation codon in the reference sequence, according to journal guidelines (www.hgvs.org/mutnomen). The initiation codon is codon 1.

* Only observed in control individuals;
† Also observed in control individuals;
§ In proximity to exon/intron boundary;
NP: breakpoints not mapped
Italic: known polymorphism

Supp. Table S4-1. *PINK1* Classic mutations [(a)]

(a) Missense, nonsense and frameshift mutations

Region	NG Numbering	cDNA Numbering	Protein Numbering	Alias	dbSNP Number	Reference
EX1	g.05164_05195del32	c.70_101del32	p.K24GfsX31	Lys24fs	-	[Chaudhary et al., 2006]
EX1	g.05188G>A	c.94G>A	p.G32R	Gly32Arg	-	[Choi et al., 2008]
EX1	g.05249C>T	c.155C>T	p.P52L	Pro52Leu	-	[Brooks et al., 2009]
EX1	g.05293C>T	c.199C>T	p.L67F	Leu67Phe	-	[Marongiu et al., 2008]
EX1	g.05297_05298GC>CT	c.203_204G>C	p.R68P	Arg68Pro	-	[Valente et al., 2004b]
EX1	g.05327C>T	c.233C>T	p.A78V	Ala78Val	-	[Kumazawa et al., 2008]
EX1	g.05369G>T	c.275G>T	p.C92F	Cys92Phe	-	[Valente et al., 2004b]
EX1	g.05376C>T	c.282C>T	p.R98W	Arg98Trp	-	[Marongiu et al., 2008]
EX1	g.05426T>G	c.332T>G	p.I111S	Ile111Ser	-	[Marongiu et al., 2008]
EX1	*g.05438A>T*	*c.344A>T*	*p.Q115L*	*Gln115Leu*	-	[Bonifati et al., 2005]
EX1	g.05465C>T	c.371C>T	p.A124V	Ala124Val	-	[Marongiu et al., 2008]
EX1	g.05467T>G	c.373T>G	p.C125G	Cys125Gly	-	[Ibanez et al., 2006]
EX1	g.05471A>C	c.377A>C	p.Q126P	Gln126Pro	-	[Prestel et al., 2008]
EX2	g.09434C>T	c.434C>T	p.T145M	Thr145Met	rs45604240	[Marongiu et al., 2008]
EX2	g.09440G>A	c.440G>A	p.R147H	Arg147His	-	[Healy et al., 2004]
EX2	g.09502G>C	c.502G>C	p.A168P	Ala168Pro	-	[Valente et al., 2004b]
EX2	g.09509T>G	c.509T>G	p.V170G	Val170Gly	-	[Toft et al., 2007]
EX2	g.09558G>C	c.558G>C	p.K186N	Lys186Asn	-	[Djarmati et al., 2006]
EX2	g.09577G>A	c.577G>A	p.G193R	Gly193Arg	-	[Weng et al., 2007]
EX2	g.09587delC	c.587delC	p.P196QfsX25	Pro196fs	-	[Kumazawa et al., 2008]
EX2	g.09587C>T	c.587C>T	p.P196L	Pro196Leu	rs35802484	[Bonifati et al., 2005]
EX2	g.09625C>G	c.625C>G	p.P209A	Pro209Ala	rs17852513	[Lee et al., 2009]
EX2	g.09626C>T	c.626C>T	p.P209L	Pro209Leu	rs34677717	[Djarmati et al., 2006]
EX2	g.09650C>A	c.650C>A	p.A217D	Ala217Asp	-	[Leutenegger et al., 2006]
EX3	g.11454A>G	c.692A>G	p.E231G	Glu231Gly	-	[Djarmati et al., 2006]
EX3	g.11466A>T	c.704A>T	p.N235I*	Asn235Ile	-	[Djarmati et al., 2006]

EX3	g.11471A>G	c.709A>G	p.M237V†	Met237Val	-	[Macedo et al., 2009]
EX3	g.11477C>T	c.715C>T	p.Q239X	Glu239Stop	-	[Hatano et al., 2004]
EX3	g.11480G>A	c.718G>A	p.E240K	Glu240Lys	-	[Rogaeva et al., 2004]
EX3	g.11498C>T	c.736C>T	p.R246X	Arg246Stop	-	[Hatano et al., 2004]
EX3	g.11493C>G	c.731C>G	p.A244G	Ala244Gly	-	[Gelmetti et al., 2008]
EX3	g.11532C>T	c.770C>T	p.T257I	Thr257Ile	-	[Marongiu et al., 2008]
EX3	g.11536C>A	c.774C>A	p.Y258X	Tyr258Stop	-	[Tan et al., 2006b]
EX4	g.16046A>G	c.787A>G	p.R263G*	Arg263Gly	-	[Rogaeva et al., 2004]
EX4	g.16061C>G	c.802C>G	p.L268V	Leu268Val	-	[Tan et al., 2005]
EX4	g.16072C>A	c.813C>A	p.H271Q	His271Gln	rs28940284	[Hatano et al., 2004]
EX4	g.16086G>A	c.827G>A	p.R276Q	Arg276Gln	-	[Marongiu et al., 2008]
EX4	g.16095G>A	c.836G>A	p.R279H	Arg279His	rs61735932	[Klein et al., 2005]
EX4	g.16097G>A	c.838G>A	p.A280T	Ala280Thr	-	[Tan et al., 2006b]
EX4	g.16146C>T	c.887C>T	p.P296L*	Pro296Leu	-	[Valente et al., 2004b]
EX4	g.16148delG	c.889delG	p.D297MfsX22	Asp297fs	-	[Kumazawa et al., 2008]
EX4	*g.16173C>T*	*c.914C>T*	*p.P305L*	*Pro305Leu*	*rs7349186*	[Healy et al., 2004]
EX4	g.16185G>A	c.926G>A	p.G309D	Gly309Asp	-	[Valente et al., 2004a]
EX4	g.16197C>T	c.938C>T	p.T313M	Thr313Met	-	[Chishti et al., 2006]
EX4	g.16208G>A	c.949G>A	p.V371I	Val317Ile	-	[Abou-Sleiman et al., 2006]
EX4	g.16211A>T	c.952A>T	p.M318L	Met318Leu	-	[Rogaeva et al., 2004]
EX5	g.17111C>T	c.965C>T	p.P322L	Pro322Leu	-	[Marongiu et al., 2008]
EX5	g.17161G>A	c.1015G>A	p.A339T	Ala339Thr	rs55831733	[Rogaeva et al., 2004]
EX5	g.17164G>A	c.1018G>A	p.A340T	Ala340Thr	rs3738136	[Valente et al., 2004a]
EX5	g.17169G>A	c.1023G>A	p.M341I	Met341Ile	rs35813094	[Lee et al., 2009]
EX5	g.17170A>G	c.1024A>G	p.M342V	Met342Val	-	[Kumazawa et al., 2008]
EX5	g.17172G>A	c.1026G>A	p.M342I	Met342Ile	-	[Nuytemans et al., 2009]
EX5	g.17186T>C	c.1040T>C	p.L347P†	Leu347Pro	rs28940285	[Hatano et al., 2004]
EX5	g.17230G>C	c.1084G>C	p.D362H*	Asp362His	-	[Rogaeva et al., 2004]
EX5	g.17246A>G	c.1100A>G	p.N367S	Asn367Ser	-	[Choi et al., 2008]
EX5	g.17252T>C	c.1106T>C	p.L369P	Leu369Pro	-	[Ibanez et al., 2006]
EX6	g.20074G>A	c.1147G>A	p.A383T	Ala383Thr	rs45515602	[Abou-Sleiman et al., 2006]
EX6	g.20080T>C	c.1153T>C	p.F385L	Phe385Leu	-	[Choi et al., 2008]
EX6	g.20084G>C	c.1157G>C	p.G386A	Gly386Ala	-	[Ibanez et al., 2006]
EX6	g.20089T>C	c.1162T>C	p.C388R	Cys388Arg	-	[Li et al., 2005b]
EX6	g.20111G>T	c.1184G>T	p.G395V	Gly395Val	-	[Marongiu et al., 2008]
EX6	g.20123C>T	c.1196C>T	p.P399L	Pro399Leu	-	[Tang et al., 2006]
EX6	g.20147G>A	c.1220G>A	p.R407Q	Arg407Gln	-	[Fung et al., 2006a]
EX6	g.20153G>T	c.1226G>T	p.G409V	Gly409Val	-	[Ibanez et al., 2006]
EX6	*g.20158G>A*	*c.1231G>A*	*p.G411S*	*Gly411Ser*	*rs45478900*	[Abou-Sleiman et al., 2006]

EX6	g.20174C>G	c.1247C>G	p.P416R	Pro416Arg	-	[Myhre et al., 2008a]
EX6	g.20177A>G	c.1250A>G	p.E417G	Glu417Gly	-	[Hatano et al., 2004]
EX7	g.20539_20561del23	c.(1252-2)_1274del23	p.?	23bp del acceptor site ex7	-	[Marongiu et al., 2007]
EX7	g.20544T>C	c.1255T>C	p.S419P	Ser419Pro	-	[Myhre et al., 2008a]
EX7	g.20569C>A	c.1280C>A	p.A427E	Ala427Glu	-	[Brooks et al., 2009]
EX7	g.20562C>T	c.1273C>T	p.P425S	Pro425Ser	-	[Rogaeva et al., 2004]
EX7	g.20580T>C	c.1291T>C	p.Y431H	Tyr431His	-	[Abou-Sleiman et al., 2006]
EX7	g.20598T>C	c.1309T>C	p.W437R	Trp437Arg	-	[Kumazawa et al., 2008]
EX7	g.20600G>A	c.1311G>A	p.W437X†	Trp437Stop	-	[Valente et al., 2004a]
EX7	g.20614T>C	c.1325T>C	p.I442T	Ile442Thr	-	[Valente et al., 2004b]
EX7	g.20641A>G	c.1352A>G	p.N451S	Asn451Ser	-	[Abou-Sleiman et al., 2006]
EX7	g.20655C>T	c.1366C>T	p.Q456X	Gln456Stop	rs45539432	[Bonifati et al., 2005]
EX7	g.20671T>G	c.1382T>G	p.L461R*	Leu461Arg	-	[Abou-Sleiman et al., 2006]
EX7	g.20680G>A	c.1391G>A	p.R464H	Arg464His	-	[Valente et al., 2004b]
EX7	g.20715G>A	c.1426G>A	p.E476K†	Glu476Lys	-	[Myhre et al., 2008a]
EX7	g.20733G>A	c.1444G>A	p.V482M	Val482Met	-	[Kumazawa et al., 2008]
EX7	g.20755T>C	c.1466T>C	p.L489P	Leu489Pro	-	[Rogaeva et al., 2004]
EX7	g.20763C>T	c.1474C>T	p.R492X	Arg492Stop	rs34208370	[Hatano et al., 2004]
EX8	g.21984C>T	c.1493C>T	p.P498L	Pro498Leu	-	[Toft et al., 2007]
EX8	g.21993G>A	c.1502G>A	p.R501Q*	Arg501Gln	-	[Abou-Sleiman et al., 2006]
EX8	g.22048delG	c.1557delG	p.K520RfsX3	Leu519fs	-	[Ibanez et al., 2006]
EX8	*g.22053A>C*	*c.1562A>C*	*p.N521T*	*Asn521Thr*	*rs1043424*	[Myhre et al., 2008a]
EX8	g.22064G>A	c.1573G>A	p.D525N†	Asp525Asn	-	[Valente et al., 2004b]
EX8	g.22064_22065insTTAG	c.1473_1574insTTAG	p.D525VfsX38	Asp525fs	-	[Rohe et al., 2004]
EX8	g.22093_22094insCAA	c.1602_1603insCAA	p.Q534_S535insQ	534_535insQ	-	[Klein et al., 2005]
EX8	g.22100G>A	c.1609G>A	p.A537T	Ala537Thr	-	[Marongiu et al., 2008]
EX8	g.22116A>G	c.1625A>G	p.N542S	Asn542Ser	-	[Kumazawa et al., 2008]
EX8	g.22138_22141delTGTG	c.1647_1650delTGTG	p.C549WfsX5	Cys549fs	-	[Ibanez et al., 2006]
EX8	g.22214T>C	c.1723T>C	p.C575R	Cys575Arg	-	[Abou-Sleiman et al., 2006]
EX8	g.22236G>T	c.1745G>T	p.X582L†	Stop582Leu	-	[Toft et al., 2007]

(b) Splice, silent and UTR variants

Region	NG Numbering	cDNA Numbering	Protein Numbering	Alias	dbSNP Number	Reference
5'UTR	g.05013G>A	c.-82G>A	p.? *	c.-82G>A	-	[Bonifati et al., 2005]
5'UTR	g.05074G>A	c.-21G>A	p.?	c.-21G>A	-	[Klein et al., 2005]
5'UTR	g.05075C>T	c.-20C>T	p.? *	c.-20C>T	-	[Bonifati et al., 2005]
EX1	*g.05283C>T*	*c.189C>T*	*p.L63*	*Leu63*	*rs45530340*	[Valente et al., 2004b]
IVS1	*g.09381A>G*	-	p.?	*IVS1-7A>G*	*rs2298298*	[Valente et al., 2004a]
EX3	g.11470A>C	c.708A>C	p.T236	Thr236	-	[Rogaeva et al., 2004]

EX4	g.16063A>G	c.804A>G	p.L268	Leu268	-	[Weng et al., 2007]
EX4	g.16096C>T	c.837C>T	p.R279	Arg279	-	[Toft et al., 2007]
EX4	g.16111C>T	c.852C>T	p.S284*	Ser284	-	[Bonifati et al., 2005]
EX4	g.16138C>A	c.879C>A	p.V293	Val293	-	[Godeiro-Junior et al., 2009]
EX4	g.16147T>G	c.888T>G	p.P296	Pro296	-	[Rogaeva et al., 2004]
EX4	g.16195G>A	c.936G>A	p.R312	Arg312	rs56200357	[Valente et al., 2004a]
EX4	g.16207C>T	c.948C>T	p.L316	Leu316	-	[Rogaeva et al., 2004]
IVS4	*g.17101G>A*	-	p.?	*IVS4-5G>A*	*rs3131713*	[Valente et al., 2004a]
EX5	g.17211A>G	c.1065A>G	p.Q355	Gln355	-	[Valente et al., 2004b]
EX5	g.17241C>T	c.1095C>T	p.S365	Ser365	-	[Valente et al., 2004b]
EX6	g.20100T>C	c.1173T>C	p.D391†	Asp391	rs45499398	[Myhre et al., 2008a]
EX6	g.20139C>T	c.1212C>T	p.Y404	Tyr404	-	[Weng et al., 2007]
EX	g.20157C>T	c.1230C>T	p.N410	Asn410	-	[Godeiro-Junior et al., 2009]
EX7	g.20549G>A	c.1260G>A	p.T420	Thr420	-	[Brooks et al., 2009]
EX7	g.20651C>T	c.1362C>T	p.Y454	Tyr454	rs56383368	[Weng et al., 2007]
IVS7	g.20791C>G	-	p.?	IVS7+14C>G	-	[Valente et al., 2004b]
EX8	g.22219A>G	c.1728A>G	p.S576*	Ser576	-	[Rogaeva et al., 2004]
3'UTR	g.22274T>A	c.*37T>A	p.?	c.*37T>A	rs686658	[Rogaeva et al., 2004]
3'UTR	g.22277G>A	c.*40G>A	p.?	c.*40G>A	-	[Rogaeva et al., 2004]

Supp. Table S4-2. *PINK1* Copy number variations [(a)]

Region	NG Numbering	cDNA Numbering	Protein Numbering	Alias	Reference
Complete gene	-	c.(?_-94)_(*2566_?)del	Complete protein	delPINK1 (NP)	[Marongiu et al., 2007]
EX4-8	-	c.(777-?)_(2566+?)del	p.?	ex4-8del (NP)	[Cazeneuve et al., 2009]
EX6-8	-	c.(1124-?)_(2566+?)del	p.?	ex6-8del (NP)	[Li et al., 2005b]
EX7	-	c.(1252-?)_(1488+?)del	p.V418_K496del	ex7del (NP)	[Camargos et al., 2009]

For all references please visit the Parkinson Disease mutation database PDmutDB (http://www.molgen.ua.ac.be/PDmutDB).
[(a)] NG numbering: NG_008164.1; cDNA numbering: NM_032409.2, protein numbering: NP_115785.1. Nucleotide numbering reflects cDNA numbering with +1 corresponding to the A of the ATG translation initiation codon in the reference sequence, according to journal guidelines (www.hgvs.org/mutnomen). The initiation codon is codon 1.
* Only observed in control individuals;
† Also observed in control individuals;
§ In proximity to exon/intron boundary;
NP: breakpoints not mapped
Italic: known polymorphism

Supp. Table S5-1. PARK7 Classic mutations [(a)]

Region	NG Numbering	cDNA Numbering	Protein Numbering	Alias	dbSNP Number	Reference
(a) Missense, nonsense and frameshift mutations						
EX2	g.06161T>C	c.29T>C	p.L10P	Leu10Pro	-	[Guo et al., 2008]
EX2	g.06210G>A	c.78G>A	p.M26I	Met26Ile	-	[Abou-Sleiman et al., 2003]
EX3	g.08695G>T	c.115G>T	p.A39S	Ala39Ser	-	[Tang et al., 2006]
EX3	g.08772G>C	c.192G>C	p.E64D§	Glu64Asp	-	[Hering et al., 2004]
EX5	*g.14281G>A*	*c.293G>A*	*p.R98Q*	*Arg98Gln*	*rs71653619*	[Abou-Sleiman et al., 2003]
EX5	g.14298G>A	c.310G>A	p.A104T§	Ala104Thr	-	[Clark et al., 2004]
EX7	g.28277A>C	c.446A>C	p.D149A	Asp149Ala	-	[Abou-Sleiman et al., 2003]
EX7	g.28302_28304delGCC	c.471_473delGCC	p.P158del	Pro158del	-	[Macedo et al., 2009]
EX7	g.28318G>A	c.487G>A	p.E163K	Glu163Lys	-	[Annesi et al., 2005]
EX7	g.28328T>C	c.497T>C	p.L166P	Leu166Pro	rs28938172	[Bonifati et al., 2003]
EX7	g.28342G>T	c.511G>T	p.A171S*	Ala171Ser	-	[Clark et al., 2004]
EX7	g.28354A>G	c.523A>G	p.K175E	Lys175Glu	-	[Nuytemans et al., 2009]
EX7	g.28366G>A	c.535G>A	p.A179T	Ala179Thr	rs71653622	[Nuytemans et al., 2009]
(b) Splice, silent and UTR variants						
Region	NG Numbering	cDNA Numbering	Protein Numbering	Alias	dbSNP Number	Reference
EX4	g.12733C>T	c.234C>T	p.G78	Gly78	rs11548937	[Abou-Sleiman et al., 2003]
IVS4	g.12759_12760insA	-	p.?	IVS4+8_9insA	-	[Tarantino et al., 2009]
EX5	g.14282G>A	c.294G>A	p.R98	Arg98	-	[Abou-Sleiman et al., 2003]
IVS5	g.14312_14322del11	-	p.?	IVS5+2_12del	-	[Hedrich et al., 2004a]
EX7	g.28311C>A	c.480C>A	p.T160	Thr160	-	[Pankratz et al., 2006b]
EX7	g.28332A>G	c.501A>G	p.A167	Ala167	rs71653621	[Abou-Sleiman et al., 2003]
EX7	g.28389T>C	c.558T>C	p.V186	Val186	-	[Hering et al., 2004]
EX7	g.28521_28522insA	c.690_691insA	p.?	c.*120insA	-	[Abou-Sleiman et al., 2003]
EX7	g.28604G>A	c.773G>A	p.?	c.*203G>A	-	[Abou-Sleiman et al., 2003]

Supp. Table S5-2. *PARK7* Copy number variations [(a)]

Region	NG Numbering	cDNA Numbering	Protein Numbering	Alias	Reference
EX1-5	g.02064_16161del14098	c.1_322del	p.?†	ex1-5del	[Bonifati et al., 2003]
EX1-5	-	c.(-129-?)_(322+?)dup	p.?	ex1-5dup (NP)	[Macedo et al., 2009]
EX5	-	c.(253-?)_(107+?)del	p.S85VfsX10	ex5del (NP)	[Djarmati et al., 2004]
EX5-7	-	c.(253-?)_(795+?)del	p.?	ex5-7del (NP)	[Hedrich et al., 2004a]

For all references please visit the Parkinson Disease mutation database PDmutDB (http://www.molgen.ua.ac.be/PDmutDB).

[(a)] NG numbering: NG_008271.1; cDNA numbering: NM_007262.4; protein numbering: NP_009193.2. Nucleotide numbering reflects cDNA numbering with +1 corresponding to the A of the ATG translation initiation codon in the reference sequence, according to journal guidelines (www.hgvs.org/mutnomen). The initiation codon is codon 1.
* Only observed in control individuals;
† Also observed in control individuals;
§ In proximity to exon/intron boundary;
NP: breakpoints not mapped
Italic: known polymorphism
